# Single-cell trajectory analysis reveals a CD9 positive state to contribute to exit from stem cell-like and embryonic diapause states and transit to drug-resistant states

**DOI:** 10.1038/s41420-023-01586-9

**Published:** 2023-08-04

**Authors:** Xi Li, Alfonso Poire, Kang Jin Jeong, Dong Zhang, Gang Chen, Chaoyang Sun, Gordon B. Mills

**Affiliations:** 1 Division of Oncological Sciences Knight Cancer Institute, Oregon Health and Science University, Portland, OR 97201 USA; 2 Department of Obstetrics and Gynecology, Tongji Hospital, Tongji Medical College, Huazhong University of Science and Technology, 430030 Wuhan, China

**Keywords:** Cancer therapeutic resistance, Tumour heterogeneity

## Abstract

Bromo- and extra-terminal domain (BET) inhibitors (BETi) have been shown to decrease tumor growth in preclinical models and clinical trials. However, toxicity and rapid emergence of resistance have limited their clinical implementation. To identify state changes underlying acquisition of resistance to the JQ1 BETi, we reanalyzed single-cell RNAseq data from JQ1 sensitive and resistant SUM149 and SUM159 triple-negative breast cancer cell lines. Parental and JQ1-resistant SUM149 and SUM159 exhibited a stem cell-like and embryonic diapause (SCLED) cell state as well as a transitional cell state between the SCLED state that is present in both treatment naïve and JQ1 treated cells, and a number of JQ1 resistant cell states. A transitional cell state transcriptional signature but not a SCLED state transcriptional signature predicted worsened outcomes in basal-like breast cancer patients suggesting that transit from the SCLED state to drug-resistant states contributes to patient outcomes. Entry of SUM149 and SUM159 into the transitional cell state was characterized by elevated expression of the CD9 tetraspanin. Knockdown or inhibition of CD9-sensitized cells to multiple targeted and cytotoxic drugs in vitro. Importantly, CD9 knockdown or blockade sensitized SUM149 to JQ1 in vivo by trapping cells in the SCLED state and limiting transit to resistant cell states. Thus, CD9 appears to be critical for the transition from a SCLED state into treatment-resistant cell states and warrants exploration as a therapeutic target in basal-like breast cancer.

## Facts

- TNBC breast cancer models have cells in multiple states including a stem cell/embryonic diapause state, a transitional state and multiple drug-resistant states.
- TNBC cells transit from stem cell-like and embryonic diapause states to drug-resistant states.
- CD9 is elevated when cells enter the transitional state and appears to be required for transit to drug-resistant states.
- A transitional state transcriptional signature but not the stem cell/embryonic diapause state signature predicts poor outcomes in TNBC.
- Knockdown of CD9 sensitizes cells to multiple therapeutic agents.

## Open Questions

- How do TNBC cells survive JQ1 treatment stress and what are the specific transcriptional states TNBC cells utilize to enter drug resistant state?
- How do TNBC cells transit among persisting states and drug-resistant states under treatment pressure of JQ1?
- What is the key modulator promoting transition among different cell transcriptional states?
- Can we target the transitional states to prevent resistance development?

## Introduction

Bromo- and extra-terminal domain (BET) inhibitors (BETi) including bromodomain 4 inhibitors (BRDi) displace BET proteins from chromatin resulting in altered oncogenic transcriptional programs and cell states that are associated with decreased tumor growth in preclinical models and clinical trials [1, 2]. However, toxicity and rapid emergence of resistance have limited their clinical implementation [3, 4].

Persister cell states represent major contributors to therapy resistance. The defining characteristics of the persister cell state are that the resistance is due to an epigenetic change (ie non-genomic) and that the resistant state returns to a drug-sensitive state following the removal of therapy. Recent data suggests that tumor cells can co-opt an embryonic diapause state, used by embryonic stem cells to survive under stress by entering a dormant state, to survive the stresses associated with cancer therapy [5–7]. A stem-like state has also been proposed to drive drug tolerance, cancer metastasis, and recurrence [8–11]. As cells exit from the stem cell-like state they can acquire more differentiated states that can contribute to worsen outcomes [12, 13]. JQ1, a potent BETi with selectivity for bromodomain 1 of BRD4, has been reported to induce expansion and self-renewal of hematopoietic stem cells [14], suggesting that the ability of JQ1 to de-differentiate cells could also contribute to stem cell enrichment and development of a drug-resistant persister cell state. Thus, the transcriptional state changes that allow cancer cells to enter and exit stem cell-like and embryonic diapause states and to transit to a drug-resistant persister cell state could represent a new class of therapeutic targets. Our current understanding of the state changes that allow entry and exit from stem cell-like and embryonic diapause states and transition to persister cells states are not sufficient to enable therapeutic targeting.

Recent studies, based on parental JQ1-sensitive (SUM149P and SUM159P) and resistant (SUM149R and SUM159R) TNBC cell lines, have explored underlying mechanisms associated with the acquisition of resistance to JQ1 [3, 15]. In these models, over time a subset of the JQ1-resistant cells reverts to a JQ1 sensitive state consistent with resistance being due to a persister cell state. Analysis of genomic changes between JQ1 resistant and sensitive populations failed to identify driver mutations that would explain the emergence of JQ1 resistance. This was supported by CRISPR and drug screens that identified a series of synthetic lethal approaches that demonstrated activity in sensitive and resistant cells but again could not be explained by genomic changes. Based on these observations, we hypothesized that epigenomic “state changes” likely underlie the acquisition of JQ1 resistance in SUM149R and SUM159R cells. We thus treated SUM149P and SUM159P as representative of JQ1 sensitive cells and SUM149R and SUM159R as representative of resistant persister cells that provide a model for exploring state changes associated with transition into resistant cell states.

Single-cell transcriptomic analysis (scRNAseq) enables visualization of cell states, transit between cell states, cellular differentiation and lineage expansion at single-cell resolution. RNA trajectory and velocity analysis by combining information from newly transcribed unspliced RNA and mature spliced mRNA allows the assignment of single cells along a dynamic trajectory represented as latent time as well as transition speed represented as velocity length [16]. To understand the changes in cell states that contribute to the development of BETi resistance, we visualized cell communities in parental and resistant SUM149 and SUM159 treated with and without JQ1. We show that stem cell-like and embryonic diapause (SCLED) cell states form a precursor for the development of JQ1-resistant states. Cells exit from the SCLED state and move to resistant states through a transitional cell state characterized by the acquisition of high CD9 expression. Interestingly, a transcriptional signature of the transitional cell state but not of the SCLED state correlates with poor outcomes in basal-like breast cancer patients, suggesting that the ability to transit between cell states is a key contributor to patient outcomes. CD9 knockdown or anti-CD9 sensitized SUM149 to multiple targeted and cytotoxic drugs in vitro, indicating a generalized role of CD9 in drug resistance. CD9 knockdown and blockade sensitized SUM149 to JQ1 in vivo by trapping cells in the SCLED state and limiting transit to resistant cell states. Thus, CD9 appears to be critical for the ability of triple-negative breast cancer cells to escape from a stem cell-like/embryonic diapause state and transition into a treatment-resistant persister cell state.

## Results

### Cells in stem cell-like and embryonic diapause transit to JQ1-resistant states in breast cancer cell lines

**Fig. 1:**
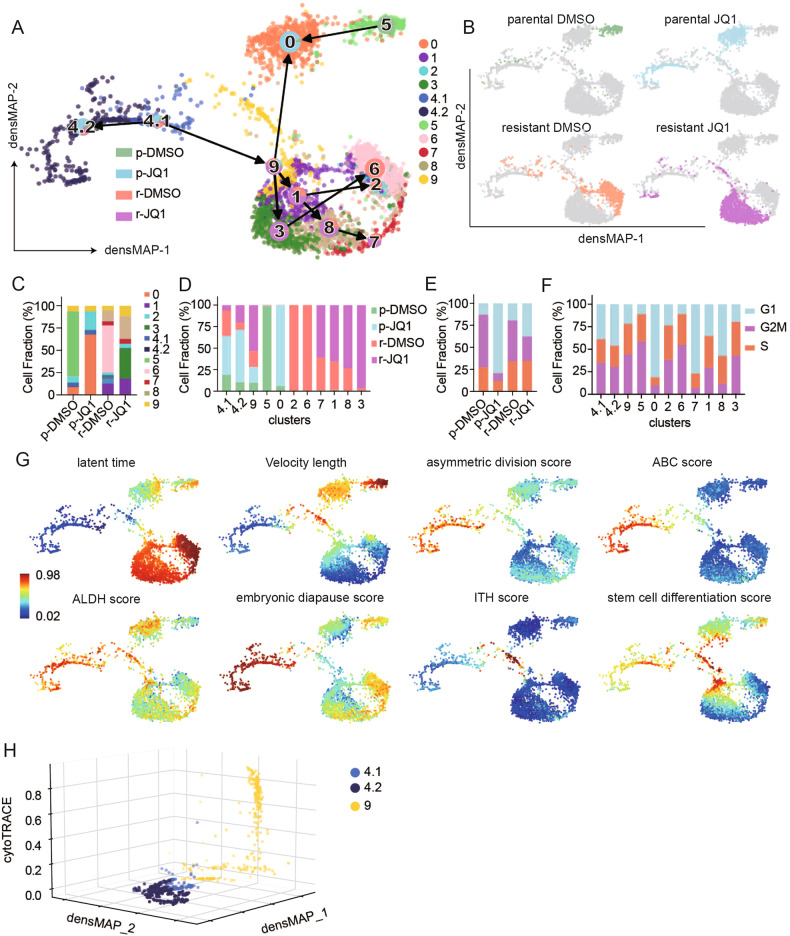
SUM149 cells in stem cell-like and embryonic diapause states transit to JQ1 resistant states. **A** Partition-based graph abstraction (PAGA) representation of Louvain community velocity clustered using latent time in scVelo were mapped on a density preserving uniform manifold approximation and projection map. (densMAP) of SUM149 cells. Each cluster community is indicated by a unique color. Frequency of cells undergoing different treatments is shown by the pie chart mapped on each cluster and bar chart displayed in (**D**). cluster0: *n* = 638, percentage of mitochondrial reads (pctMT) = 8.51. cluster1: *n* = 362, pctMT = 7.12. cluster2: *n* = 55, pctMT = 11.04. cluster3: *n* = 457, pctMT = 9.67. cluster4.1: *n* = 95, pctMT = 42.18. cluster4.2 *n* = 293, pctMT = 47.16. cluster5: *n* = 341, pctMT = 8.38, cluster6: *n* = 518, pctMT = 11.04. cluster7: *n* = 121, pctMT = 8.77. cluster8: *n* = 444, pctMT = 8.81. cluster9: *n* = 262, pctMT = 6.09. **B** The origin of each cell from SUM149P and SUM149R and treatment conditions are mapped on the densMAP in **A**. **C** Fraction of cells of each cluster in each treatment condition. **D** Fraction of cells of each treatment condition in each cluster. **E** Fraction of cells in different cell cycle phases in each treatment condition. **F** Fraction of cells in different cell cycle phases in each cluster. **G** Single cells are colored by normalized latent time, velocity length, embryonic diapause score, ABC score, ALDH score, asymmetric division score, ITH score and stem cell differentiation score mapped on to **A**. Scale is at the left bottom of the latent time panel. **H** CytoTRACE score is embedded in three-dimensional visualization of single cells from cluster 4 and cluster 9.

SUM149R and SUM159R represent a model of epigenome-mediated cell state transition to a drug-resistant persister cell state [15]. We thus obtained and evaluated scRNA-seq datasets from SUM149 and SUM159 parental and resistant cells using a dynamic trajectory analysis approach (scVelo) to identify cells states associated with the transition to drug resistance. To resolve heterogeneity during the transition to a resistant state, a density-preserving uniform manifold approximation and projection (densMAP) was used to visualize community relationships (aka Louvain clusters, [17]). DensMap incorporates single-cell transcriptional variability as well as the local density of data spaces to provide a more representative visualization of cell state alterations [17]. We identified 10 distinct densMAP clusters or communities in parental and resistant SUM149 and 9 clusters in parental and resistant SUM159 with and without treatment with JQ1 (Fig. 1A and Fig. S1A).

Similar to previous analysis [15], after batch effect removal (Methods), the resultant clusters demonstrated markedly different frequencies of SUM149 cells from the different treatment conditions (SUM149P: vehicle, SUM149P: JQ1, SUM149R: vehicle, SUM149R: JQ1) (Fig. 1A–D) with marked separation of the majority of the SUM149P and SUM149R cells. Cluster 0 and 5 were almost exclusively parental cells incubated with and without JQ1, respectively (<1% contamination with SUM149R cells). Similarly, clusters 1,2,3,6,7, and 8 were almost exclusively SUM149R (<1% contamination with SUM149P) with clusters 6 and 2 being almost exclusively vehicle treated and clusters 1,3,7,8 being dominantly SUM149R treated with JQ1 (Fig. 1A–D). In marked contrast, cluster 4.1, 4.2, and 9 contained a mixture of treatment naïve as well as post-treatment sensitive and resistant cells with cluster 4.1 and 4.2 being enriched for JQ1-treated SUM149P and cluster 9 enriched for SUM149R (Fig. 1A–D). In the presence of JQ1, there was a marked decrease in the proportion of SUM149P that were in cell cycle with a more modest decrease in SUM149R (Fig. 1E, F). The mixed clusters (4.1, 4.2, and 9) all had a relatively high frequency of cells in cycle.

Trajectory directionality was then overlaid on the densMAP velocity clustering. On JQ1 treatment, cluster 4.1 transited to cluster 4.2 (blue) that does not seem to be able to transit further and to cluster 9 (yellow) that appears to be an intermediary between cluster 4.1 and all other clusters (except for cluster 5) including the SUM149R clusters. Cluster 5, which was dominantly vehicle-treated SUM149P, appears to transit on JQ1 treatment to cluster 0 that does not transit further. Interestingly, clusters 1,3,7,8 that are dominantly SUM149R treated with JQ1 appear to transit to clusters 6 and 2 that are dominantly vehicle-treated SUM149R. Since SUM149R was maintained in JQ1 [15], the JQ1 treated condition is the “normal” condition and the vehicle-treated condition is the “new” condition; thus, transit to cluster 6 and 2 is the expected directionality.

We subsequently focused on clusters 4.1 and 9 that were common to both SUM149P and SUM149R treated with and without JQ1 and that appeared to represent cells with the potential to produce the remaining clusters and in particular the resistant clusters (cR, clusters 1,2,3,6,7, and 8).

Cluster 4.1 that had low latent time and velocity length appeared to be upstream of the other clusters (Fig. 1A, G). The low-velocity length is consistent with cluster 4.1 representing a relatively stable state. As indicated in Fig. 1G, cluster 4.1 (and also 4.2) had signatures consistent with a stem cell-like population (asymmetric cell division, ABC, and ALDH scores) as well as a high embryonic diapause score (Fig. 1G, [7, 18]). We thus designated cluster 4.1 as stem cell-like population/embryonic diapause (SCLED). Interestingly, both stem cell-like cells and embryonic diapause cells have been proposed to mediate drug resistance [6, 7, 13].

We designated cluster 9, which was positioned between cluster 4.1 and the cR clusters (Fig. 1A, G), as the “transitional” cluster. A subset of cells in cluster 9 had a high-velocity length, high percentage of cells in cycle, high intratumoral heterogeneity (ITH) score and a high stem cell differentiation score (GO: 0048863) (Fig. 1G). The ITH score is consistent with transitional cluster 9 being highly plastic and the high stem cell differentiation score is consistent with cluster 9 being an intermediary population between SCLED cluster 4 and the remaining clusters. Supporting cluster 9 as a transitional cluster, CytoTRACE [19] showed that the majority of the cells in cluster 9 have a much higher “development potential” than cells in cluster 4.1 and 4.2 (Fig. 1H). In contrast, the resistant clusters and SCLED cluster have low-velocity scores and likely represent more stable states.

The densMAP clusters of SUM159 recapitulated those of SUM149 with cluster 0 and 5 being almost exclusively SUM159P, and clusters 1,2,3,6, and 7 being almost exclusively SUM159R (Figure S1A–F). Again, similar to SUM149, SUM159 cluster 4 and 9 contained cells from all treatment conditions (Figure S1A–F). Trajectory directionality of SUM159 was also similar to that of SUM 149 with cluster 4 (blue) appearing to transit to cluster 9 (yellow) which represents a transitional state between cluster 4 and the SUM159R clusters. Again, similar to SUM149, the mixed SUM159 clusters (4 and 9) had a relatively high frequency of cells in cycle. Cluster 4 SUM159 had higher asymmetric cell division, ABC, and embryonic diapause scores consistent with a SCLED designation (Fig. 1G and Figure S1G). As in SUM149, the velocity length of SUM159 cluster 4 was consistent with it being relatively stable with limited entry and exit and the velocity length of cluster 9 consistent with it being relatively unstable and rapidly transiting.

The similarity in clusters and processes between SUM149 and SUM159 is further supported by the overlap in differentially expressed gene signatures (DEG) consisting of the top 15 DEGs for each SUM149 and SUM159 cluster compared to all other clusters (Figure S1H). However, cluster 2 and 7 in SUM159 did not have a strong equivalent in SUM149. SUM159 cluster 6 and 3 had representation from multiple different clusters in SUM149 that constitute the SUM149 cR cell population.

Thus, the overall structure and transitions of clusters and in particular, SCLED cluster 4 being a precursor to the remaining clusters and cluster 9 being a transitional cluster between cluster 4 and JQ1 resistant clusters is consistent between SUM149 and SUM159. This suggests that the transitional cluster could represent an interesting therapeutic target.

We next sought to determine whether the SCLED and transitional cell state were unique to the SUM149 and SUM159 models or generalizable across cell lines. We thus trained a prediction model with SUM149 scRNA data (Fig. 1A), and then classified individual cells in a pan-cancer scRNA dataset of 196 cell lines [20] to a SCLED or cluster 9-like subtype (from Fig. 1A) using scPred, which is designed to accurately classify single cells [21]. We found that SCLED and cluster 9-like cells pre-existed in multiple treatment naïve cell lines (Figure S1I, J). Indeed, approximately half of the cell lines contained 1%–5% cluster 9-like cells (95% confidence) similar to the levels in SUM149 and SUM159 lines (Fig. 3B). Interesting, in the 196 cell line set [20], similar to SUM149, the cluster 9 signature and to a lesser degree the SCLED state was associated with an increased ITH score (Figure S1K).

### The transitional but not the SCLED cluster is associated with metastatic potential and worsened patient outcomes

**Fig. 2:**
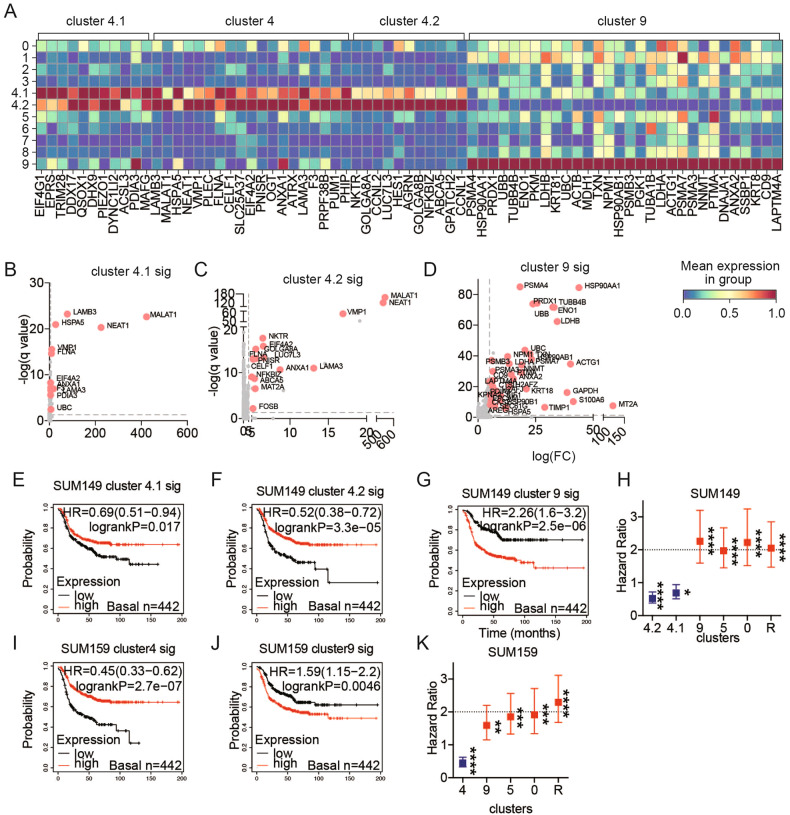
The transitional but not the SCLED cluster is associated with metastatic potential and worsened patient outcomes. **A** Heatmap of normalized expression (log_2_(TPM + 1)) of top 30 DEGs across cluster 4.1, cluster 4.2 and cluster 9 in SUM149. DEGs present in both cluster 4.1 and cluster 4.2 are indicated as cluster 4. **B**–**D** Volcano plot shows differentially expressed genes in cluster 4.1, 4.2 and cluster 9 in SUM149. Each pink dot represents a gene with Log_2_(FC) higher than 5 as well as adjusted p-value less than 0.01. **E**–**G** Kaplan–Meier curve of relapse-free survival in patients with basal-like breast cancer (*n* = 442) based on DEGs with log_2_FC ≥ 5 and adjusted *p*-values < 0.01 for cluster 4.1 (**E**), cluster 4.2 (**F**), cluster 9 (G) of SUM149. **H** Hazard ratio predicted by Kaplan–Meier curve of relapse-free survival in patients with basal-like breast cancer (*n* = 442) based on DEGs with log_2_FC ≥ 5 and adjusted *p*-values < 0.01 for cluster 4.1, cluster 4.2, cluster 9, cluster 5 and cluster 0 and cluster R in SUM149. Significance levels were determined by log-rank test. *p < 0.05, **p < 0.01, ***p < 0.001, ****p < 0.0001. **I**, **J** Kaplan–Meier curve of relapse-free survival in patients with basal-like breast cancer (*n* = 442) based on DEGs with log_2_FC ≥ 5 and adjusted *p*-values < 0.01 for cluster 4 (**J**) and cluster 9 (**K**) in SUM159. **K** Hazard ratio predicted by Kaplan–Meier curve of relapse-free survival in patients with basal-like breast cancer (*n* = 442) based DEGs with log_2_FC ≥ 5 and adjusted *p*-values < 0.01 for cluster 4, cluster 9 and cluster R in SUM159. Significance levels were determined by log-rank test. *p < 0.05, **p < 0.01, ***p < 0.001, ****p < 0.0001.

We used DEGs to explore features defining the SCLED (cluster 4) and transitional cluster (cluster 9), (Fig. 2A–D). The greater number of upregulated DEGs in transitional cluster 9 is consistent with increased cluster 9 ITH. In SCLED cluster 4, two long non-coding RNA (lncRNA, MALAT1 and NEAT1) that are implicated in stem cell renewal [22] were strikingly up-regulated (Fig. 2A–C). This is consistent with the stem cell-like characteristics of cluster 4 noted in Fig. 1H. Transitional cluster 9 had higher levels of heat shock proteins (HSP90AA1, HSP90AB1) (HSP) as well as proteasome proteins (PSMA4, PSMA7, PSMA3, PSMB3) that have been implicated in apoptosis, cell proliferation and differentiation (Fig. 2A, D, Fig. S2A, B). A subset of DEGs from SUM149 SCLED cluster 4 and transitional cluster 9 were also overexpressed in SUM159 cluster 4 and 9, respectively (Fig. S2A). Interestingly, a protein-protein interaction network derived from 6 overlapping DEGs of SUM149 and SUM159 transitional cluster 9 was associated with HSP chaperon-mediated autophagy and cell cycle progression (Fig. S2B).

We next explored whether the SUM149 cluster signatures were associated with outcomes in basal-like breast cancer using microarray data from the KM plotter database (442 patients). Surprisingly, a signature comprised of DEGs with Log_2_(FC) higher than 5 as well as adjusted p-value less within SUM149 SCLED cluster 4 was associated with a favorable outcome (Table S2). Interestingly, the same embryonic diapause transcriptome was associated with a statistically significantly improved outcome in basal breast cancer (Fig. S2C) and lung adenocarcinoma (Fig. S2D) consistent with the outcomes associated with the cluster 4 transcriptome suggesting that entry into the embryonic diapause state is associated with a lineage-specific effect on outcomes. However, when we assessed a previously described embryonic diapause transcriptome that was associated with a poor outcome in colorectal cancer [7], it was also associated with a worsened outcome in lung squamous cell carcinoma, and gastric, and ovarian cancer (Fig. S2D). In support of an ability to exit from a stem cell-like or embryonic diapause state and transit to a resistant state being associated with a poor outcome, the signatures of SUM149 transitional cluster and resistant clusters were associated with a statistically significant worsened survival in basal-like breast cancer patients (Fig. 2E–H). To further test the association of the cell states with patient outcomes, we repeated the analysis with SUM159 signatures (Table S3). SUM159 SCLED cluster 4 signature was once again associated with an improved outcome, whereas the SUM159 transitional cluster 9 signature and the resistant cluster signature were associated with statistically significant worsened outcomes (Fig. 2I–K). The data suggests that an ability to transit (transitional cluster 9) from a stem cell or embryonic diapause state (SCLED cluster 4) to a drug resistant state (cluster cR) rather than the stem cell or embryonic diapause state per se is associated with a worsened outcome in basal-like breast cancer.

**Fig. 3:**
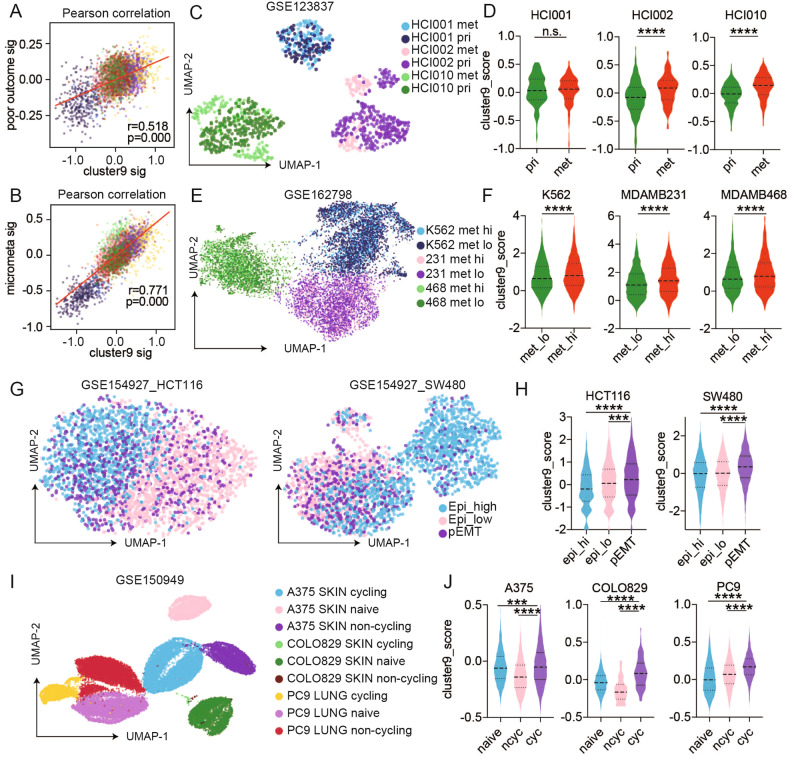
The association of cluster 9 signature and the feature of plasticity, micrometastasis, partial EMT and cycling persisters was characterized in several scRNAseq and scATACseq datasets. **A** Scatter plot of Pearson correlation of cluster 9 signature (signature of cluster 9 as above) and a signature of poor outcome in single cells. Cells are colored based on Fig. 1A. **B** Scatter plot of Pearson correlation of cluster 9 signature (signature of cluster 9 as above) and micrometastasis signature in single cells. Cells are colored based on Fig. 1A. 231: MDA-MB-231, 468: MDA-MB-468. **C** Uniform manifold approximation and projection map (UMAP) of 3 TNBC PDX tumor cells. Each condition is indicated by a unique color. **D** Violin plot expression of cluster 9 signature in each indicated condition from datasets in **A**. **E** Uniform manifold approximation and projection map (UMAP) of 3 TNBC cell lines. Each condition is indicated by a unique color. **F** Violin plot expression of cluster 9 signature in each indicated condition from datasets in **C**. **G** Uniform manifold approximation and projection map (UMAP) of 2 colon cancer cell lines. Each condition is indicated by a unique color. **H** Violin plot expression of cluster 9 signature in each indicated condition from datasets in **E**. **I** Uniform manifold approximation and projection map (UMAP) of 3 skin cancer cell lines and 1 lung cancer cell line. Each condition is indicated by a unique color. **J** Violin plot expression of cluster 9 signature in each indicated condition from datasets in **G**.

Based on the association of the cluster 9 signature with worsened patient outcomes, we sought potential underlying mechanisms. The SUM149 transitional cluster 9 signature demonstrated concordance with a previously published signature that predicts a poor outcome in basal-like breast cancer [23, 24] (Fig. 3A). Further SUM149 transitional cluster 9 signature was highly concordant with a previously published micrometastasis signature [25] consistent with metastasis from the primary lesion being the key cause of a worsened outcome in basal-like breast cancer (Fig. 3B). The cluster 9 signature was also significantly enriched in 2 of 3 lung metastatic compared to primary TNBC PDX (Fig. 3C, D) [25]. scATAC-seq datasets of lung and breast cancer cell lines selected for metastatic propensity [26] also demonstrated a statistically significant association with the cluster 9 signature (Fig. 3E, F). In two colon lines, the cluster 9 signature was associated with a partial EMT signature [27], which has been implicated in metastatic potential (Fig. 3G, H) [28]. Finally, the cluster 9 signature was significantly associated with cycling persister cells in both skin and lung cancer cell lines (Fig. 3I, J) [29].

Taken together, the data support a concept wherein the ability to exit from a SCLED state and transit to resistant cell states through a cluster 9-like state is associated with a worsened outcome in multiple cancers. It also further demonstrates that cells with the cluster 9 signature are present in multiple different model and cell line systems and not restricted to SUM149 and SUM159.

### CD9 expression is increased in transitional cluster 9

**Fig. 4:**
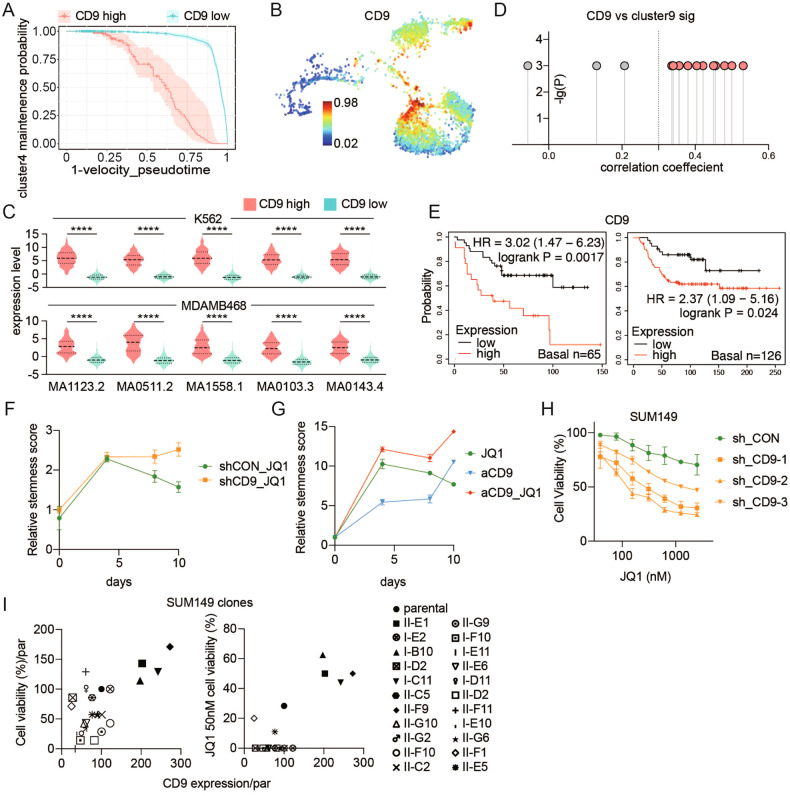
CD9 expression is increased in transitional cluster 9 and regulates JQ1 resistance in SUM149. **A** Likelihood of cells remaining in the SCLED cluster based on CD9 expression. **B** CD9 levels of individual cells are mapped on the graph in Fig. 1A. **C** Scatter plot shows Pearson correlation of average CD9 expression and cluster 9 signature in each dataset in Fig. 3. **D** Violin plot shows indicated motif activity of CD9 high cluster and CD9 low cluster in K562 and MDAMB468 cells. **E** Kaplan–Meier curve of relapse-free survival in patients with basal-like breast cancer (*n* = 65 or *n* = 165) based on low or high CD9 protein split on median level. Significance is determined by log-rank test. **F** Changes of in vitro stemness score of SUM149 transfected with shCON or shCD9-1 lentivirus treated with JQ1 (100 nM) for 96 h, 8 days and 10 days. In vitro stemness markers included SETD6, NANOG, POU5F1, MALAT1, and NEAT1 were assessed by RT-PCR and in vitro stemness score calculated as un weighted sum of relative fold change of each gene compared to shCON cells treated with DMSO and shCD9 cells treated with DMSO, respectively. Data represent SEM of three replicates. **G** Changes of in vitro stemness score of SUM149 treated with JQ1 (100 nM), anti-CD9 and combination for 96 h, 8 days and 10 days. **H** Cell viability assessed by prestoblue of SUM149 transfected with shCON, shCD9-1, shCD9-2, and shCD9-3 lentivirus and treated with indicated doses of JQ1 for 96 h. **I** Scatter plot of correlation of cell growth assessed by prestoblue and CD9 expression (left). Scatter plot of cell sensitivity to JQ1 and CD9 expression (right).

We next attempted to identify processes associated with SUM149 leaving SCLED cluster 4, entering transitional cluster 9 and transiting to JQ1-resistant clusters (cR). We utilized Cox-regression to identify DEGs from cluster 9 that correlated with movement along the velocity pseudotime axis. The use of velocity pseudotime avoids the identification of events associated with changes in cell cycle and renewal that correlate best with the latent time axis [30]. Of DEGs associated with cluster 9, CD9 was most highly upregulated upon exit from SCLED cluster 4 and entry into transitional cluster 9 in velocity pseudotime (HR: 31, *p* = 1.2 × 10^−19^, Fig. 4A, B, Supplemental Table S4). CD9 was co-expressed with several targets (EP300, PSMA3 and PSMD13) previously shown to be synergistic with JQ1 and was mutually exclusive with targets (CDKN1A) associated with resistance to JQ1 (Fig. S3A–C) [15]. It was also mutually exclusive with the embryonic diapause signature genes (ALDH6A1 and CTSL) (Fig. S3C) [7]. In K562 and MDAMB468 scATAC-seq datasets, CD9 enriched clusters demonstrated higher chromVAR scores of SNAI1 (MA1558.1), TWIST (MA1123.2), RUNX2 (MA0511.2), ZEB1 (MA0103.3), and SOX2 (MA0143.4) consistent with higher transcription plasticity in CD9 high cells [31–35] (Fig. 4C). CD9 expression alone was able to recapitulate the cluster9 signature in 10 out of 14 individual scRNAseq datasets analyzed in Fig. 3 as well as an additional human TNBC scRNA-seq dataset (Fig. 4D, [36]). CD9 RNA levels did not predict outcomes in all TNBC subtypes, but were sufficient to predict a poor outcome in basal-like 2 breast cancer patients, a therapy-resistant subtype (Figure S3D) [37]. CD9 protein levels were sufficient to recapitulate the prognosis associated with the cluster 9 transitional cell transcriptome in predicting shorter relapse-free survival in two TNBC cohorts that included CD9 protein levels (Fig. 4E) [38, 39].

To determine whether the increase in CD9 contributed to exit from SCLED cluster 4, we assessed the effects of CD9 knockdown in stable SUM149P shRNA clones and anti-CD9 treatment on effects of JQ1 on stem cell markers (SETD2, SETD6, NANOG, POU5F1) and the top two DEGs in SCLED cluster 4 (MALAT1, NEAT1) that are associated with stem-cell renewal [22]. The relative level of these markers (SCLED stemness score) was increased on day 4 of JQ1 treatment of SUM149P (Fig. 4F, G). In control cells, the stemness score decreased on day 8 and 10 of JQ1 treatment consistent with JQ1 causing cells to exit SCLED. In contrast, the high stemness score persisted in both shCD9 knockdown and CD9 antibody-treated SUM149P (Fig. 4F, G) suggesting that the SCLED state is maintained in the absence of CD9 activity. This is consistent with the concept that CD9 expression is required for SUM149 cells to exit SCLED cluster 4, enter transitional cluster 9 and then progress to JQ1 resistant states. Stable knockdown of CD9 with shRNA in SUM149P also sensitized SUM149P to JQ1 (Fig. 4H) but importantly sensitized cells to JQ1 in additional TNBC cell lines (Fig. S3E–G) providing support for the concept that CD9 plays a generalized role in JQ1 responsiveness in TNBC. Furthermore, after co-expression of a shRNA targeting the CD9 3’ untranslated region (UTR) and a CD9 expression construct that lacks the CD9 3’ UTR and is thus rescues cells with the CD9 shRNA, CD9 positive SUM149 cells were significantly enriched by JQ1 treatment (Figure S3H). Based on the different levels of CD9 in SUM149P cells, we performed single-cell cloning and were able to isolate clones with low and high CD9 levels. Interestingly cells with elevated CD9 levels had higher proliferative rates than cells with low CD9 levels (Fig. 4I). Strikingly, the four SUM149 clones with elevated CD9 levels were markedly resistant to JQ1 (Fig. 4I and Fig. S4A–C). Consistent with high CD9 and drug resistance due to a state change rather than a genomic aberration, over time the clones tended to lose their differential CD9 expression levels and return to expression and drug sensitivity levels similar to parental cells, a characteristic associated with persister cells (Fig. S4D, E).

JQ1 dissociates BRD4 from chromatin, allowing it to move to the cytosol [40]. We thus assessed the effect of JQ1 on BRD4 location in SUM149P clones with different CD9 levels. In JQ1-treated SUM149P, cells that lacked nuclear BRD4 were not in cycle based on Ki67 levels (Fig. S5A–D). There was, however, a small population of JQ1-treated SUM149P cells with nuclear BRD4 and retained Ki67 expression (Fig. S5A–D). Strikingly, in SUM149P clones with high CD9, BRD4 nuclear localization and Ki67 staining were maintained in the majority of JQ1-treated cells. In contrast, JQ1 excluded BRD4 from the nucleus and decreased Ki67 in SUM149P clones with low CD9 levels (Fig. S5A–D). Furthermore, stable knockdown of CD9 with shRNA was associated with a marked decrease in nuclear BRD4 and Ki67 (Fig. S5A–D). Interestingly, CD9 knockdown was associated with micronuclei formation and mitotic dysfunction (Figure S5A–D). Thus, elevated CD9 levels are associated with resistance to the effects of JQ1 on BRD4 nuclear localization and thus DNA binding.

Together the data support a mechanistic role for CD9 in response to JQ1 in SUM149P.

### CD9 mediates resistance to multiple but not all drugs

**Fig. 5:**
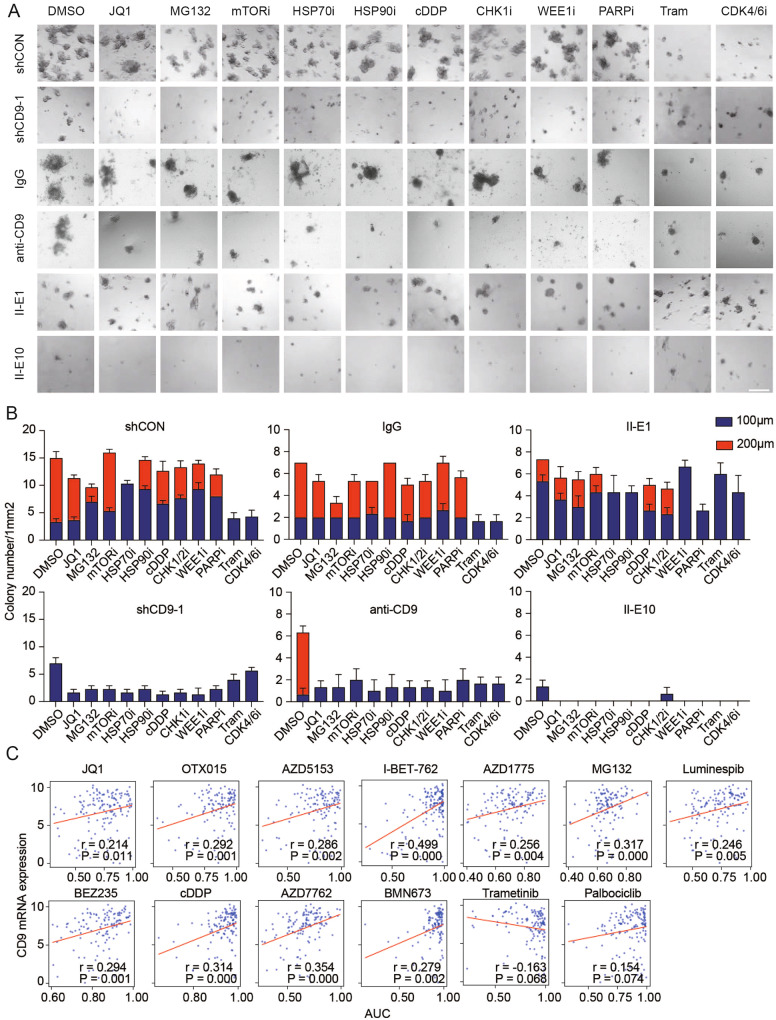
CD9 contributes to resistance to multiple drugs. **A** Representative light microscopy images of 3D cultured cell lines treated with indicated drug for 96 h. (JQ1 = 100 nM, MG132 = 400 nM, mTORi = 200 nM, HSP70i = 10 μM, HSP90i = 8 nM, cDDP=300 nM, CHK1i = 1 nM, WEE1i = 60 nM, PARPi=600 nM, Tram=500 nM, CDK4/6i = 1 μM). Data are representative of three replicates. Scale bars: 200 μm. **B** Bar charts of colony numbers with indicated diameter with indicated treatments in **A**. C Scatter plots of Pearson correlation of CD9 expression and indicated drug sensitivity in cancer cell lines from CTRPv2. Each dot presents a cell line.

Based on the role of CD9 in mediating state transition, we assessed the effects of CD9 levels using SUM149P clones and CD9 knockdown as well as anti-CD9 on responses to a suite of targeted agents and found that decreased CD9 activity was associated with increased sensitivity to a proteasome inhibitor (MG132), kinase inhibitors (mTORi), DNA damage checkpoint inhibitors (PARPi, CHK1i, WEE1i), heat shock family inhibitors (HSP70i, HSP90i) and chemotherapy (cisplatin) (Fig. 5A, B). Interestingly, of the drugs tested, CD9 knockdown or CD9 inhibition did not increase sensitivity to MEK or CDK4/6 inhibitors in SUM149P (Fig. 5A, B). We subsequently explored the CTRPv2 database and found that CD9 levels were associated with many of the drugs assessed in Fig. 5 (Fig. 5C) as well as additional BET inhibitors (AZD5153, OTX015, I-BET-762), a combined mTOR PI3K inhibitor (BEZ225) and a PARP inhibitor (BMN673). Thus, CD9 expression contributes to resistance to multiple but not all drugs.

### CD9 inhibition increases JQ1 sensitivity in vivo

**Fig. 6:**
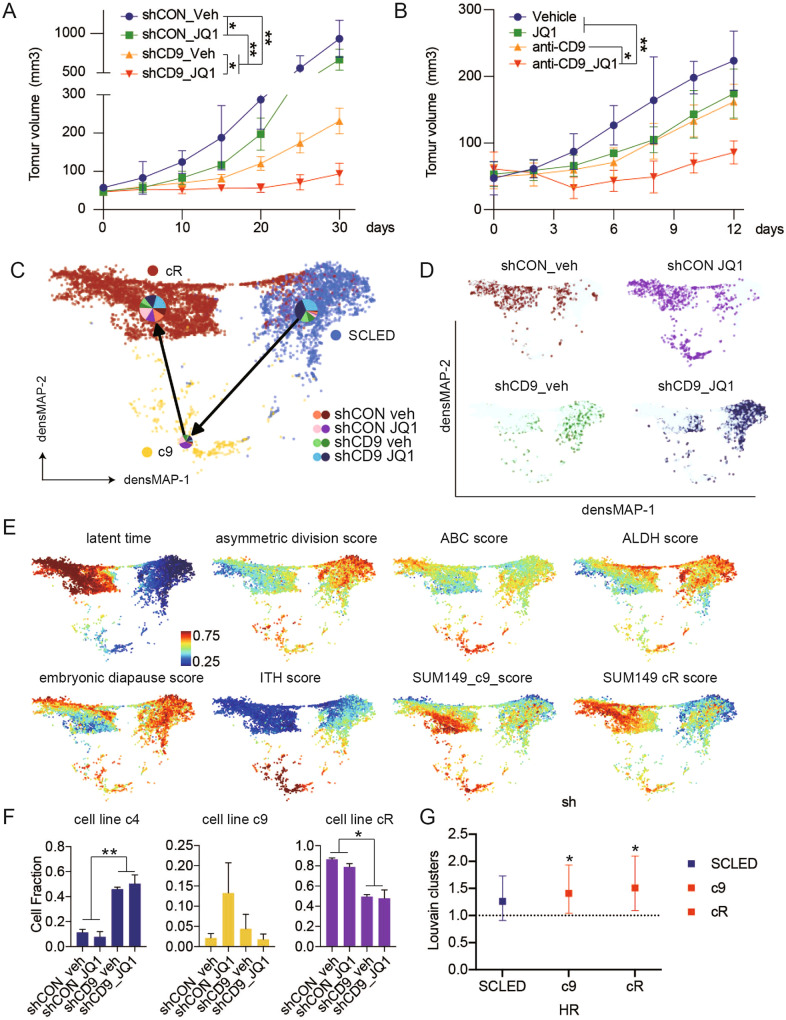
SUM149 cells are sensitive to the combination of JQ1 and CD9 inhibition. **A** Tumor growth curves of indicated groups. SUM149 with or without stable CD9 knockdown were injected into mice. Five mice were treated with vehicle or JQ1 (25 mg/kg intraperitoneal daily) for 30 days. Tumor volume was measured with calipers. **B** Tumor growth curves of indicated groups. Five mice were treated with vehicle and IgG (1 mg/kg, every second day), JQ1 (10 mg/kg intraperitoneal daily), anti-CD9 antibody (1 mg/kg, every second day) or JQ1 combined with anti-CD9 antibody for 30 days. Tumor volume was measured with calipers. **C** scRNA data from tumors collected at 30 days was assessed and visualized using densMAP as described in Fig. 1A. scPred trained on data from Fig. 1 was used to provide cell cluster identity. Frequency of each treatment is shown by pie chart mapped on each cluster. **D** Cell distribute of each indicated treatment in densMAP. **E** Single cells are colored by normalized latent time, asymmetric division score, ABC score, ALDH score, embryonic diapause score, ITH score and cluster9 signature score (c9) and resistant cluster signature score (cR) mapped onto (**C**). Scale is at the left bottom of the latent time panel. **F** Each cell in the in vivo analysis was assigned to a SUM149 cluster using scPred trained on data from Fig. 1A and frequency determined. **G** Hazard Ratio predicted by Kaplan–Meier curve of relapse-free survival in patients with basal-like breast cancer (*n* = 442) based on DEGs with log_2_FC ≥ 5 and adjusted *p*-values < 1e−9 for clusters in (**C**) in SUM149. Significance was determined by log-rank test. *p < 0.05.

In order to ascertain the role of CD9 in JQ1 resistance in a tumor model, we determined the effect of CD9 knockdown and blockade with an anti-CD9 antibody on response to JQ1 in SUM149 xenografts. CD9 knockdown SUM149P formed smaller tumors than those from shCON cells suggesting that CD9 may contribute to tumor growth independent of response to JQ1 potentially through effects of proliferation noted above (Fig. 6A). As indicated in Fig. 6A, JQ1, at the doses used, JQ1 had modest effects on growth of shCON SUM149P cells. In contrast, growth of CD9 knockdown SUM149P was controlled by JQ1 during the 30-day treatment period (Fig. 6A). Consistent with the effects of CD9 knockdown, anti-CD9 treatment combined with low doses of JQ1 (1/5 of dose used in a previous study) [15] markedly controlled tumor growth (Fig. 6B). The effect on growth in the mammary fat pad was recapitulated by tumor weight and tumor size at the end of the study (Fig. S6A, B).

We subsequently performed scRNAseq on SUM149P tumors isolated from mice at the end of the study. Prolonged treatment of SUM149P with JQ1 in vivo (30 days) has the potential to induce JQ1 resistance that could reflect characteristics of the SUM149R cell line. scPred, which is designed to accurately classify single cells [21], was trained on the SUM149 cell line clusters in Fig. 1A. scPred identified 4 major clusters (Fig. S6C, Fig. 6C, D). Trajectory analysis combined with densMAP was done with cell subtypes classified by scPred. The transition of cells from the SCLED cluster into cluster 9 and subsequently JQ1 resistant clusters was recapitulated in shCD9 xenograft tumors (Fig. 6C, E). CD9 knockdown increased the number of cells in the SCLED state with a decrease in the number of cells in cluster 9 and the resistant cell clusters (Fig. 6F). This is consistent with trapping of cells in the SCLED cluster. potentially contributing to the decreased tumor growth induced by the combination. While a number of targets reported to be synergistic with JQ1 [15] were upregulated by shCD9, they were markedly downregulated by the combination of shCD9 and JQ1 potentially contributing to the decreased tumor growth induced by the combination (Fig. S6E). MYC was modestly downregulated by JQ1 monotherapy and similar, to the other targets, upregulated by shCD9. However, both MYC and two Hallmark MYC target transcriptomes were markedly downregulated by the combination of JQ1 and shCD9 (Fig. S6E).

When we explored the association of signatures of different clusters identified in vivo with patient outcomes, we found once again that signature derived from SCLED was associated with an improved outcome whereas cluster 9 and cR signatures were associated with worsened patient outcomes (Supplemental Table S5, Fig. 6G).

The data is consistent with a model wherein CD9 in the transitional cluster being required for cells to transit from the SCLED state to a JQ1 resistant state.

### Proteasome function mediated by CD9 is essential for surviving JQ1

**Fig. 7:**
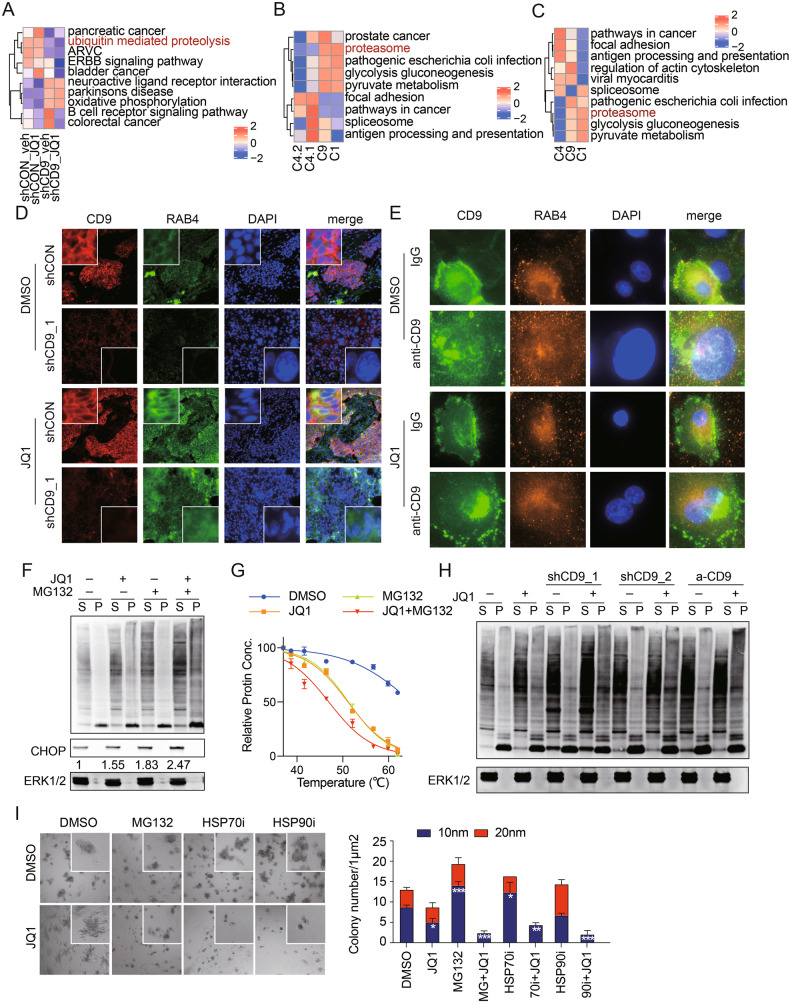
CD9 contributes to endosome and proteosome function. **A** GSVA enrichment scores of KEGG pathways in scRNA Seq of xenograft tumors from Fig. 6. **B** GSVA enrichment scores of top 50 DEGs for clusters 4.1, 4.2, 9 and 1 in SUM149. **C** GSVA enrichment scores of top 50 DEGs for clusters 4, 9 and 1 in SUM159. **D** Representative immunofluorescence images of indicated protein expression in indicated SUM149 cell lines (JQ1 = 100 nM). Data are representative of three replicates. **E** Representative immunofluorescence images of indicated protein expression in SUM149 cell lines treated with JQ1 (100 nM), anti-CD9 and combination. Data are representative of three replicates. **F** Representative western blot of data from one of three independent experiments of indicated protein in SUM149 cell line treated with indicated treatments (JQ1 = 100 nM, MG132 = 200 nM) for 96 h. **G** Whole-proteome thermal stability curve of SUM149 cell line treated with indicated treatments for 96 h. Data represent SEM of three replicates. **H** Representative western blot of indicated protein of indicated cell lines (JQ1 = 100 nM, anti-CD9 = 1 μg/μl). Data are representative of three experiments. **I** Representative light microscope images of 3D cultured SUM149 cell line treated with indicated drugs (JQ1 = 100 nM, MG132 = 400 nM, HSP70i = 10uM, HSP90i = 8 nM) for 96 h (left). Bar chart of colony numbers with indicated diameter with indicated treatments (right). Data represent SEM of three replicates. *p < 0.05, **p < 0.01, ***p < 0.001.

To explore how CD9 knockdown sensitized to JQ1 in vivo, we assessed pathway profiles across the 4 treatment conditions using GSVA. Interestingly, ubiquitin-mediated proteolysis was decreased in shCD9 tumors and further decreased in shCD9 tumors treated with JQ1 (Fig. 7A). Given the increase in the number of cells in the SCLED cluster and a decrease in the number of cells in cluster 9 in shCD9 tumors in vivo, the decrease in ubiquitin-mediated proteolysis is consistent with the increase in ubiquitin-mediated proteolysis in cluster 9 in SUM149 (Fig. 7B) and SUM159 (Fig. 7C). Furthermore, in the 196 cell line data set noted above [20], the cluster 9 signature correlated with a proteosome signature (Fig. S7A, B).

We subsequently explored the effects of JQ1, shCD9 and anti-CD9 treatment on the endosomal compartment. RAB4, a marker of early endosomes, was distributed in cytoplasm of SUM149P and shCON SUM149P (Fig. 7D, E). After JQ1 treatment, shCON SUM149P colonies lost their circumscribed characteristics and demonstrated complex morphology with spreading. RAB4 was markedly upregulated in JQ1-treated shCON SUM149P including the spreading regions of the colonies (Fig. 7D), while in 2D culture a clear co-localization of RAB4 and CD9 was observed in JQ1-treated SUM149P (Fig. 7E). Interestingly following JQ1 treatment, there was an association between CD9 and RAB4 as indicated by the yellow color. In both shCD9 and anti-CD9 treated SUM149P, cell morphology was altered with marked changes in nuclear morphology including larger size and fused nuclei (Fig. 7E). Although RAB4 expression was upregulated by JQ1 treatment, the distribution was abnormal consistent with loss of CD9 or anti-CD9 disrupting early endosome function.

In support to the importance of endosome function in JQ1 resistance, JQ1 treatment of SUM149 increased both soluble (S) and insoluble (P) ubiquitin with a much greater effect when MG132 was added to prevent degradation of ubiquitinylated proteins in the proteosome (Fig. 7F). There was a modest increase in CHOP consistent with activation of an unfolded protein response (Fig. 7F). While both JQ1 and MG132 decreased protein thermal stability, JQ1 and MG132 together have a greater effect on protein sensitivity to thermal stress (Fig. 7G). Furthermore, in SUM149P, shCD9 and anti-CD9 increased soluble ubiquitin and increased the effects of JQ1 on total and insoluble ubiquitin implicating CD9 in proteosome activity (Fig. 7H).

We subsequently determined the effect of targeting HSP90 (HSP90i) and HSP70 (HSP70i) as well as proteosome activity (MG132) on colony formation in 3 breast cancer cell lines, two pancreatic cell lines, 1 ovarian cancer cell line and 1 colorectal cell line in 2D culture (Fig. S7C). Strikingly inhibition of HSP90, HSP70, or the proteosome sensitized each line to JQ1. To more closely mimic the in vivo context, we assessed the effects of the inhibitors on the activity of JQ1 in 3D culture. In the absence of the inhibitors, in SUM149P JQ1 decreased the number of small colonies (10 nm) but had no effect on larger colonies (20 nM, Fig. 7I). Strikingly, despite HSP90i, HSP70i and MG132 having minimal activity on their own, they markedly sensitized SUM149P to JQ1 with a complete loss of larger colonies including those with a more aggressive morphology and a marked decrease in smaller colonies. Together, these data support the concept that protein stability contributes to the ability of SUM149 cells to survive the effects of JQ1.

## Discussion

Shu et al have performed a number of studies to identify mechanisms and therapeutic opportunities associated with acquisition of resistance to BET inhibition with JQ1 based on studies of JQ1-sensitive SUM149P and SUM159P and JQ1-resistant SUM149R and SUM159R. Their studies have identified differences and therapeutic opportunities between the JQ1 sensitive and resistant cells. Although there were genomic differences between JQ1 sensitive and resistant cells, they did not explain the differences in JQ1 sensitivity nor the altered responses in CRISPR and drug screening assays [15] suggesting that this is a model of epigenomically related resistance mediated by persister cells. We thus explored whether state changes as assessed by trajectory analysis could explain the transition of JQ1-sensitive SUM149P and SUM159P to JQ1-resistant SUM149R and SUM159R and importantly whether these state changes would represent new therapeutically tractable processes.

We visualized cell communities in parental and JQ1-resistant SUM149 and SUM159 treated with and without JQ1 using densMAP onto a UMAP representation. A cluster that contains independent populations of stem cell-like and embryonic diapause cell (SCLED) states appears to be upstream of a transitional cluster that contains a subset of rapidly transiting cells and a drug-resistant cluster. Cells exit the SCLED state and move through a transitional state to JQ1-resistant persister cell states. These states were observed in multiple cells lines in addition to the original SUM149 and SUM159 models supporting generalizability of the process.

scRNAseq and associated RNA velocity analysis provide an opportunity to understand both the subtypes and potential state changes in single cells. However, the relatively new velocity analysis approaches are confounded by potential technical artifacts present in the approaches used to deconvolute scRNA-seq data, the quality of the data and the assumptions underlying the derivation of trajectories of single cells based on the frequency of spliced and unspliced RNA [41]. DensMap that incorporates single-cell transcriptional variability as well as the local density of data space to provide a more representative visualization of cell state alterations [17] was used to mitigate some of the challenges with conventional RNA velocity analysis. Further, the identification of similar state and state transitions in data sets from SUM149, SUM159 and SUM149 shCD9 xenografts strengthens the confidence in the output of the RNA velocity analysis. The ability to map the states and state transitions in the in vitro SUM149 scRNA data to the in vivo scRNA data with scPred provides further support of the RNA velocity analysis. Importantly, the apparent originating state in the RNA velocity analysis was composed of cells with stem cell like and embryonic diapause-like states that expressed both the expected transcriptomes and markers associated with these states. Consistent with cluster 4 being the originating cluster, both stem cell like and embryonic diapause states are considered to be precursors to more differentiated states.

While cells from the in vivo model mapped with scPred onto the states identified in the in vitro studies, there were a number of cells that were not mapped by scPred. The trajectory of these cells and their role in resistance to JQ1 will require further investigation. However, they likely represent a novel cell type engendered by the combination of CD9 knockdown and treatment with JQ1 and further emphasize the plasticity of the SUM149 model under therapeutic challenge.

Interestingly, a signature of the transitional cell state, but not a signature of the SCLED state, correlated with poor outcomes in basal-like breast cancer patients suggesting that the ability to transit between cell states is a key contributor to patient outcomes. The transitional cell signature was also highly correlated with a micrometastasis signature, which could contribute to the association with outcomes as metastatic ability is a key determinant of patient outcomes. Metastasis requires that cells be able to transit between epithelial mesenchymal transition (EMT) and MET. The observation that the signatures derived from SCLED cluster were associated with an improved outcome in breast cancer was consistent in independent analysis of signatures derived from the SUM149 and SUM159 basal-like breast cancer models including the in vivo model giving greater confidence in the conclusions. Previous analysis of stem cell-like and embryonic diapause signatures [7, 12] have suggested that these are associated with a poor patient outcome in colorectal cancer. Our analysis of the previously derived embryonic diapause signature confirmed the association with a poor outcome in colorectal cancer [7]. The embryonic diapause transcriptome was also associated with a worsened outcome in lung squamous cell carcinoma, and gastric, and ovarian cancer. However, the same embryonic diapause transcriptome was associated with a statistically significantly improved outcome in basal breast cancer and lung adenocarcinoma. This suggests that the signatures are associated with lineage-specific effects that could be related to the intrinsic characteristics of the lineages, the mutational patterns associated with the lineages and also with the therapy approaches used in each lineage. The demonstration that a cluster 9 signature was associated with a worsened outcome in TNBC strongly supports the contention that this cluster is required for the full manifestation of drug resistance and further that it could represent a novel therapeutic target. Indeed, we demonstrated that targeting this cluster through knocking down or inhibition the main DEG associated with the cluster CD9 markedly decreased tumor growth and sensitized cells to an array of targeted and chemotherapy agents.

We used velocity pseudotime to identify DEGs associated with transit through transitional cluster 9. The CD9 tetraspanin was the DEG most highly associated with velocity pseudotime (HR: 31, *P* = 1.2 × 10^−19^). Both knockdown of CD9 and CD9 antibody treatment combined with JQ1 resulted in improved tumor control and also a marked increase in response to a suite of targeted and chemotherapy agents. However, these effects were specific to a number of different targets, as CD9 knockdown did not alter sensitivity to MEK and CDK4/6 inhibitors. The difference in sensitivity to different targeted agents remains to be explained. Knockdown of CD9 was associated with a loss of normal endosome function as indicated by changes in localization and structure of RAB4 intracellular structures. Knockdown of CD9 was also associated with changes in ubiquitination and proteosome function. This could contribute to the altered sensitivity to JQ1 as the effect of CD9 knockdown on JQ1 sensitivity was recapitulated by treatment with MG132. These results are compatible with those of Shu et al that demonstrated that a HSP90 inhibitor and MG132 as well as CRISPR of ubiquitination-related genes SPOP, UBE2M, CUL3 and USP14 demonstrated synthetic lethality with JQ1 [15].

CD9, a member of the tetraspanin superfamily, shows wide cellular and tissue distribution and is involved in cell motility, proliferation and metabolism in both immune cell and tumor cells [42, 43]. CD9 protein is found at the membrane and is associated with a wide range of physiological and biological functions including vesicular fusion, endocytosis and exosome biogenesis. CD9 inhibition has also been reported to attenuate extracellular vesicle uptake [44]. In addition to proliferation and exosome function in tumor cells, CD9 is also broadly expressed by different immune cell types with an important role in shaping both anti-tumor immunity and pro-tumor immunity depending on the different types of immune cells present in the tumor niche [45].

CD9 has been proposed to have anti- and pro-tumor capability in different tumor lineages [43, 46, 47]. In breast cancer studies, CD9 also has been reported to be involved in tumor invasion and in inhibition of tumor progression that would be expected to have opposite effects on outcomes [48, 49]. Notably, even in a single-cell line, the effect of CD9 silencing has proven controversial with proliferation promotion and inhibition being observed in MDA-MB-231 after CD9 silencing in two different studies [50, 51]. We found that CD9 protein levels were sufficient to predict a worsened outcomes in two cohorts of basal-like breast cancer patients but that CD9 mRNA in the same samples did not predict outcome. This could in part be due to discrepancies between mRNA and protein levels that we have reported previously [52, 53]. However, in the basal like 2 subset (also known as basal-like immunosuppressed) that is associated with the worst outcome, RNA levels were able to predict patient outcomes. Thus, it is possible that RNA and protein levels of CD9 do identify an ability to transition to a drug-resistant state at least in this subtype of breast cancer. Consistent with this model, both CD9 knockdown and anti-CD9 markedly increased sensitivity to combinations of JQ1 with a number of targeted and therapeutic agents in multiple cell lines in vitro. Perhaps more importantly, inhibition of CD9 in xenograft models with low doses of JQ1 that did not demonstrated toxicity in the murine models was associated with marked tumor control. As CD9 has been implicated in both anti-tumor immunity and pro-tumor immunity [45], the effects of blocking CD9 in immune-competent mice requires further exploration prior to implementation into clinical trials.

Thus, our data suggests that targeting CD9 could revitalize the opportunities for BET family inhibitors in solid tumor both by increasing their efficacy and also by decreasing the dose of BET inhibitors required to mediate optimal effects. This contention may apply to suite of targeted and chemotherapy agents in addition to BET family inhibitors. The ability to increase efficacy while decreasing toxicity and thus widening the therapeutic index is especially attractive. These preclinical data provide support for further exploration and potential development of CD9 as a therapeutic target.

## Materials and methods

### Cancer cell lines

Breast cancer cell line MDA-MB-231 and MDA-MB-468 were obtained from ATCC and cultured at 37 °C under 5% CO_2_ in DMEM with 10% (vol/vol) fetal bovine serum. SUM149 was a gift from Dr. Laura Heiser and cultured at 37 °C under 5% CO_2_ in F12 with 5% fetal bovine serum supplemented with 250ul insulin, 50ul EGF and 5 ml HEPES. Pancreatic cell line Pa02C and Pa16C were gifts from Prof. Anirban Maitra (Division of Pathology/Lab Medicine, The University of Texas MD Anderson Cancer Center) and cultured at 37 °C under 5% CO_2_ in RPMI-1640 with 10% fetal bovine serum. Ovarian cancer cell line HeyA8 was from MD Anderson Cancer Center Characterized Cell Line Core and was cultured at 37 °C under 5% CO_2_ in RMPI-1640 with 10% fetal bovine serum. CT26 was obtained from ATCC and cultured at 37 °C under 5% CO_2_ in DMEM with 10% fetal bovine serum. Cells were fingerprinted before use with short tandem repeat (STR) testing.

### Xenograft assays

For xenograft assays, female NSG (NOD.Cg-Prkdcscid Il2rgtm1Wjl/SzJ) mice at 6 weeks of age were purchased from the Jackson Laboratory. Animal experiments were performed by X. Li in Animal Facility, Knight Cancer Institute, Oregon Health & Science University according to the IACUC approved protocol: Combination Therapy Targets Adaptive Resistance in Cancer (TR01_IP00002062) following AALAC guidelines. Mice were housed 5 to a cage with *ad libitum* access to food and water in 20 °C, 40–50% humidity and a 12-h light/12-h dark cycle. Animal numbers of each group were calculated by power analysis and animals were randomly assigned to groups for each experiment. For shCD9 xenograft model, 2 × 10^6^ SUM149 cells transfected with shCON or shCD9 lentivirus and suspended in 50 μL of culture medium/Matrigel Growth Factor Reduced Basement Membrane Matrix, Phenol Red-Free (Corning, CLS356231) in a 1:1 ratio were orthotopically injected into the mammary fat pad. After 14 days, mice were assigned to treatment groups (*n* = 5) based on randomization of tumor size. Mice were treated with vehicle or JQ1 (25 mg/kg, intraperitoneally, daily, Selleckchem, #S7110) for 30 days. For anti-CD9 and JQ1 combination assay, 2 × 10^6^ SUM149 cells were injected as described above. After 14 days, mice were assigned as described above. Mice were then treated with vehicle and IgG (1 mg/kg, intraperitoneally, every second day), JQ1 (10 mg/kg), CD9 antibody (ALB6, IM0117, Beckman Coulter, 1 mg/kg, every second day) or combination for 12 days. Tumors were measured by calipers every 3 days. Tumor volume was calculated according to a modified ellipsoid formula *V* = 1/2 × (Length × Width^2^). Mice were isoflurane anesthetized and tumors were collected and fixed overnight in 10% formalin, stored in 70% ethanol followed by paraffin embedding, 5μm-sectioning and hematoxylin and eosin staining, or single-cell suspension generation for scRNAseq at the end of treatment.

### Antibodies and Inhibitors

Antibodies used for Western blotting were ubiquitin (P4D1, Santa Cruz Biotechnology, #C0821), HSP90 (Cell Signaling Technology, #4874), CHOP (Cell Signaling Technology, #2895S). Antibodies used for immunofluorescence were RAB4 (EPITMICS, #2632-1), CD9 (TS9, Abcam, #ab58989), ERK1/2 (Cell Signaling Technology, #4695). Antibodies used for cytometry were CD9 (C-4, Santa Cruz Biotechnology, #J0819), along with Calcein violet AM (Invitrogen, #65-0854-39), Propidium Iodide (PI) (Invitrogen, 00-6990-50). Antibodies used for treatment were CD9 (Abcam, # ab58989). The proteasome (MG132), HSP90 (NVP-AAUY922), HSP70 (VER155008), PARP (Olaparib), BRD4 (JQ1), CHK1 (Prexasertib), WEE1 (AZD1775), MEK (Trametinib), CDK4/6 (Palbociclib) inhibitors were purchased from Selleckchem and were of the highest quality available. cDDP was purchased from TSZ CHEM.

### Membrane protein extraction

Membrane protein was extracted by Subcellular Protein Fractionation Kit for Cultured Cells (#78840, ThermoFisher) according to the manufacturer’s instructions. Briefly, first, cell pellet was added to Cytoplasmic Extraction Buffer (CEB) and incubated at 4 °C for 10 min with gentle mixing. Then, after centrifugation at 500 × *g* for 5 min, cytoplasmic protein in the supernatant was separated from the pellet that included membrane proteins. Ice-cold Membrane Extraction Buffer (MEB) containing protease inhibitors was added to the pellet. After vortexing for 15 s, the tube was incubated at 4 °C for 10 min with gentle mixing. After centrifuging at 5000 × *g* for 5 min, membrane protein was localized in the supernatant.

### Cellular thermal shift assays for protein stability

Protein stability was measured by thermal shift assays as described [54]. Briefly, 10^6^ cells in 1 mL PBS supplemented with 2 × protease inhibitors were lysed by 3 freeze-thaw cycles in liquid nitrogen with heating and vortexing briefly after each thawing. Cell suspension lysates were cleared by centrifugation (13,000 rpm for 30 min at 4 °C). Cell suspension lysates were divided into eight 100 μL aliquots, which were then heated in a gradient thermocycler at 37, 38.7, 41.6, 46.4, 52.1, 56.7, 59.9, 62 °C for 10 min. Protein concentrations were measured by BCA.

### Insoluble ubiquitin aggregates and cell fractions

Isolation of insoluble ubiquitin aggregates was performed as described [55]. Briefly, cells were incubated in stringent lysis buffer (25 mM Tris, 150 mM NaCl, 1% NP-40, and 1% sodium deoxycholate supplemented with 2X protease inhibitors) on ice for 15 min and then sheared by passage through a 23 G needle. Lysates were cleared by centrifugation (13,000 rpm for 15 min at 4 °C) and supernatant transferred to a new tube. Pellets were washed 3 times with cold PBS with 0.1% Triton X-100 and 2X protease inhibitors. Insoluble pellets were dissolved in Laemmli sample buffer (Bio-Rad, #1610747). Protein concentrations of soluble fractions were determined by BCA. Soluble fraction volumes were adjusted to the same protein concentration. Finally, equal volumes of soluble and insoluble fractions were separated by western blotting.

### Generation of CD9 Knockdown and CD9 rescue breast cancer cells lines

shRNA (shCD9_1 #TRCN0000296954, shCD9_2 #TRCN0000291711, shCD9_3 #TRCN0000296958, shCON #SHC201) were purchased from Sigma Aldrich. Lentivirus were packaged in 293 T cell line by cotransfected into 293 T cells with Lentiviral Packaging Mix (#SHP001, Sigma Aldrich). 72 h after co-transfection, 293 T medium with packaged lentivirus was harvested. SUM149, MDAMB231 and MDAMB468 cells were transfected with 293 T medium containing lentivirus by spinning (1000 × g) for 2 h at 25 °C. Cells were selected in puromycin. SUM149 transfected with shCD9_3 which targets the CD9 3’ untranslated region (UTR) was transfected with CD9-mGFP (0.5 ng/μL, Addgene, #182864) that lacks the CD9 3’ untranslated region to rescue CD9 expression in SUM149 CD9 knockdown cells.

### Software used in this study

SCANPY, scVelo, Cell Ranger v6.0.0, GSVA, Survival R, FlowJo, samtools, CytoTrace, ImageJ, Prism (v8) were used. All statistical analyses were done by GraphPad Prism software. Non-normally distributed data was compared using the Mann–Whitney test. Wilcoxon matched-pairs signed rank test was used for paired data. Comparison among multiple groups was done by one-way Anova.

### Single-cell RNAseq data analysis

Single-cell RNAseq data generated by 10xGenomics was processed by Cell Ranger count pipeline (v6.0.0) with command argument: “include introns”. Output BAM files were further processed by Velocyto to generate LOOM files containing UMI of spliced RNA, unspliced RNA and ambiguous as separate matrices with the default parameters. Cells with human hashtag oligo reads were included for further analysis. Single-cell sample demultiplexing was done by unsupervised Louvain clustering of cell multiplexing oligo matrix and the Louvain community with exclusively unique cell multiplexing oligo captured being assigned to different treatment groups. Cells with fewer than 2000 genes detected (UMI > 4000) or with more than 30% UMIs from mitochondrial genes were excluded. Doublets were removed by Scrublet with the expected doublet rate of 6 and doubletScore larger than 95%. Filtered data were then normalized and scaled by SCANPY and scVelo to remove unwanted source of variance such as batch effects and cell cycle effects. Unsupervised clustering was performed by Louvain cluster module in SCANPY. DEGs between clusters were obtained by Wilcoxon rank-sum test. FDR was used to correct for multiple testing. Asymmetric division score (GO:0008356), embryonic diapause score, stem cell differentiation score (GO: 0048863), (ATP binding cassette) ABC score and (aldehyde dehydrogenase) ALDH score were calculated with score gene module in SCANPY with the gene list indicated in Supplemental Table S1. Density-preserving dimensionality reduction was done by densVis [17] package.

### Trajectory analysis

RNA velocity analysis was done by scVelo[30] with dynamic mode. The calculated RNA velocity vectors were embedded to low-dimension space by Partition-based graph abstraction (PAGA) module in scVelo package. CytoTRACE score was calculated by CytoTRACE R package [19]. Calculated CytoTRACE score was then embedded with UMAP into a higher dimensional graph.

### Cell state classification

scPred, a supervised classification-based automated cell type annotation tool that applies support vector machines (SVMs) [21] was used for cell subtype annotation. To provide additional information to trajectory analysis done by scVelo, SUM149 scRNA-seq matrix with only highly variable genes (HVGs) imputed by scVelo and the velocity cluster information from Fig. 1 were used as training matrix for scPred with “MDA” for a mixture discriminant analysis training model. Once the model was trained and evaluated, the trained model was applied to classify cell state from the CD9 xenograft dataset. In order to maintain a single normalization method as well as keep the imputed information from scVelo, the cell state classification was applied to the CD9 xenograft dataset after imputation.

### Pseudo-survival analysis

Cluster 9 DEGs expression were split into two high and low group using a cut off of average expression of each gene. In order to avoid embryonic diapause cell-cycle effects, the association between each of the cluster 9 DEG events and velocity pseudotime was assessed by the Cox-regression model by Survival in R. The results are presented as hazard ratios with 95% confidence intervals. The proportional hazards were visualized by inspecting graphs of the cumulative baseline functions against pseudo-survival time (1-velocity pseudotime).

### Public data analysis

Single-cell RNAseq fastq files of SUM149, SUM149R, SUM159, and SUM159R cell lines treated with JQ1 were obtained from GSE131102 and processed as described above. Cell and gene filter threshold were set described with minor modification as cells with more than 55% UMIs from mitochondrial genes [15]. The filtered data were then normalized and scaled by using SCANPY and scVelo. The batch effect was removed by regress out total counts, total genes and mitochondrial gene percentage. Single-cell RNAseq data of TNBC patients was obtained from GSE118390. Fastq files were aligned against GRCh38 human reference including transcripts with STAR (v2.7.6a). Output BAM files were then processed by Velocyto to generate LOOM files containing UMI of spliced RNA, unspliced RNA and ambiguous as separate matrices. Cells with fewer than 1000 genes were excluded from further analysis. 868 epithelial cells were filtered for further Louvain clustering and stem differentiation score analysis. Processed scRNA-seq datasets of TNBC PDX models (GSE123837), colon cancer cell lines (GSE154927), skin cancer cell lines and lung cancer cell line (GSE150949) were utilized. Processed scATAC-seq datasets (GSE162798) of lung cancer cell line and breast cancer cell lines were utilized. CD9 and treatment co-dependent data of cell lines were obtained from Cancer Therapeutics Response Portal (CTRPv2).

### Single-cell ATAC-seq data analysis

The cellranger processed ATAC-seq output was used as the input to Signac package v1.7.0 [56]. Cells with lower strength of nucleosome-binding pattern or lower transcription start site enrichment score or lower total number of fragments in peaks (<3000) or lower fraction of fragments in peaks (<15) or higher percentage of reads in ENCODE-blacklisted genomic regions (<0.05) were filtered out from further analysis. Normalization and dimensionality reduction were performed using Signac with default parameters. Transcript information was pulled from EnsDb databases. Transcription factor motif enrichment was performed using ChromVAR version 1.16.0 [57].

### Supplementary information

supplemental information

## Data Availability

This study did not generate custom code, and the wrapped code used in the study is freely available from https://github.com/xili3367/JQ1_CD9. The software used in this article: ImageJ (https://imagej.nih.gov/ij/), Prism (http://www.graphpad.com/faq/viewfaq.cfm?faq=1362), FlowJo (https://www.flowjo.com/), Cellranger (http://10xgenomics.com), Velocyto (https://github.com/velocyto-team), R (https://www.r-project.org/; RRID: SCR_001905), SCANPY (https://github.com/theislab/scanpy), scVelo (https://github.com/theislab/scvelo), densVis (https://github.com/hhcho/densvis/tree/master/densmap). Data of SUM149, SUM149R, SUM159, and SUM159R cell lines are available: GSE131102. Data of pan-cancer cell lines are available: GSE157220. Data of lung metastasis of TNBC PDX are available: GSE123837. Data of scATAC-seq of breast cancer and leukemia cell lines are available: GSE162798. Data of scRNA-seq of colon cancer cell lines are available: GSE154927. Data of scRNA-seq of SKIN and LUNG cancer cell lines are available: GSE150949. Data of scRNA-seq of human TNBC tumors are available: GSE118390. scRNA-seq data of SUM149 xenograft tumors can be found under the accession number GSE221183.
