# Supplementary material for: Single-cell trajectory analysis reveals a CD9 positive state to contribute to exit from stem cell-like and embryonic diapause states and transit to drug-resistant states
Source: Cell Death Discov. 2023 Aug 4;9:285. doi: 10.1038/s41420-023-01586-9 (PMC10403509; doi:10.1038/s41420-023-01586-9)
Supplement: Supplementary file 1 — supplemental information [file 41420_2023_1586_MOESM1_ESM.docx]

**Supplemental information**

**Supplemental methods**

Immunofluorescence staining

Before treatment, cells were seeded in 8 well chamber slides (Thermo Fisher Scientific). After deparaffinization and rehydration, slides were subjected to antigen retrieval in citrate buffer pH 6 (Sigma, C9999-100ML) for 20 minutes and in eBioscience IHC Antigen Retrival Solution pH 9 buffer (Invitrogen, #00-4956-58) for 15 minutes in a steamer. Blocking solution (Abcam, ab126587) was applied for 15 minutes at room temperature. After washing three times with PBS, primary antibody was incubated in PBS supplemented with 5% Normal Goat Serum (NGS) overnight at 4℃ in a moist chamber. After three times washing with PBS, secondary antibody was applied for 1 hour at room temperature. Samples were stained with DAPI (Invitrogen, #D1306) for 5 minutes in room temperature. Samples were mounted with 50% (v/v) glycerol (Fisher Scientific, #329032). Immunofluorescence was observed and photographed by Yokogawa CSU-X1. ZEN (black edition) and ImageJ was used for image analysis.

Western blotting

Cells were lysed in RIPA buffer supplemented with cOmplete, Mini, EDTA-free Protease Inhibitor Cocktail (Roche, #05892970001) and PMSF (Sigma, #PMSF-RO) for 30 minutes, sonicated on ice and cleared by centrifugation (13,000 rpm for 15 minutes at 4℃). Protein concentration was determined using bicinchoninic acid assay (BCA) method. Protein was then adjusted to equal concentration. Proteins were separated by gel electrophoresis and transferred to polyvinylidene difluoride membranes. After incubation for 30 min at RT with 5% w/v BSA in Tris-buffered saline supplemented with 0.1% Tween20 (TBST) membranes were then probed with the desired primary antibody at 4℃ for overnight and secondary antibody at room temperature for 1 hour. Bands were visualized using Pierce ECL Western Blotting Substrate (Thermo Fisher, #32209). ERK1/2 was used as loading control.

qRT-PCR

Cells were treated as described before isolating RNA with NucleoSpin RNA Plus for RNA purification with DNA removal column (#740984) and cDNA synthesis with SuperScript IV VILO Master Mix with ezDNase Enzyme (Invitrogen, #11766050). Gene expression was detected using PowerUp SYBR Green Master Mix (Thermofisher, #A25742) by QuantStudio 3 Real-time PCR system. Expression was normalized to the expression of internal control ATP5B. Values represent an average of three replicates.

Proliferation assays

SUM149 parental cell line, SUM149 transfected with shCON and SUM149 transfected with shCD9 lentivirus were plated to 96-well plates at 2000 cells per well. Cell lines were treated as described. After 3-day treatment, cells were washed three times with PBS and incubated with fresh complete medium supplemented with 10% (vol/vol) prestoblue at 37℃ under 5% CO_2_ for 3 hours. Absorbance was read at 570nm by Varioskan LUX. After subtracting the media blank, viability was calculated following normalization to DMSO. Values represent an average of three independent experiments.

Colony formation assays

Cell lines were plated into 6-well plates at 500 cells per well. Cell lines were treated as described. After 14-day treatment, cells were washed three times with PBS and fixed with 4% formaldehyde solution for 5 min before staining with crystal Violet 0.5% solution (Fisher Science, #S25275B). Colony number was counted by ImageJ. Images represent one of three replicates.

Single cell RNAseq library generation

SUM149 xenograft tumors were disaggregated by gentleMACS kits (Miltenyi Biotec, #130-096-730), and incubated in TotelseqA hashtag antibodies (Biolegend, #394601, #394603, #394605, #394607, #394609, #394611, #394613, #394615) following the manufactural instructions. Live cells were isolated by EasySep Dead Cell Removal (Annexin V) Kit (STEMCELL Technologies, #17899). Single cell suspensions were processed according to 10xGenomics scRNAseq sample preparation protocol (Chromium Single Cell 3’ v3.1 Reagent Kit, 10xGenomics). 1000 cells were targeted for each sample.

| **Table S1, related to Figure 1. Summary of the gene lists used to calculate indicated score** | |
| --- | --- |
| Name | Marker genes |
| Asymmetric division score | ACTR3, ACTR2, RGS14, RAB10, ETV5, FGF13, ASPM, GOLGA2, STRA8, ING2, INSC, PAX6, POU5F1, PARD3, SOX5, TEAD3, WNT9B, ZBTB16, DOCK7, ARHGEF2 |
| stem differentiation score | PDCD6, EPOP, PSME3, CDK6, PSMD14, SEMA3A, YAP1, N4BP2L2, SEMA6C, SEMA6B, SEMA4F, SEMA4D, SEMA4B, SEMA3C, BATF, CFL1, NUDT21, PWP1, SOX21, POU6F2, TRIM6, SP7, PSMB11, MSI2, OSR1, H1-8, PSMA8, PRICKLE1, GSC, NKX2-5, RDH10, ACE, DHX36, JAG1, EDN1, EDN3, EDNRA, EDNRB, EFNB1, A2M, TAPT1, ERBB4, ERCC2, ESR1, ESRRB, RBM24, SEMA3D, FGFR2, MTF2, FOXC1, FOXC2, KDM4C, PSME4, FN1, FOLR1, ZNF281, CORO1C, FRZB, ABL1, NELFB, GATA1, GATA2, GATA3, GATA4, GATA6, GBX2, GREM1, GDNF, GPM6A, SETD2, DNMT3L, SOX8, NRG1, HIF1A, FOXA1, HNRNPU, HOXA7, HOXB4, HES1, HSPA9, HTR2B, CYP26C1, RBPJ, PDX1, ISL1, JARID2, KIT, HES5, LAMA5, LIF, SHC4, LMO1, LMO2, LRP6, LTBP3, MIR146A, TACSTD2, EPCAM, TAFA1, SMAD4, MEF2C, MEOX1, KITLG, ASCL1, KMT2A, FOXO4, MSX1, MSX2, MYB, NFE2L2, NOTCH1, NRTN, NTF4, OSM, PAX2, YTHDF2, PDGFRA, CDK12, PITX2, SOX18, PUS7, SEMA4C, OCIAD1, PEF1, SOX6, LMBR1L, KDM3A, MAPK1, MAPK3, EIF2AK2, METTL3, PSMA1, PSMA2, PSMA3, PSMA4, PSMA5, PSMA6, PSMA7, PSMB1, PSMB2, PSMB3, PSMB4, SEMA3G, PSMB5, PSMB6, PSMB7, PSMB8, PSMB9, PSMB10, PSMC1, PSMC2, PSMC3, PSMC4, PSMC5, PSMC6, PSMD1, PSMD2, PSMD3, PSMD4, PSMD5, PSMD7, PSMD8, PSMD9, PSMD10, PSMD11, PSMD12, PSMD13, PSME1, PSME2, SEMA6A, PTN, SEMA4G, PTPRC, OVOL2, BCHE, KLHL12, REST, RET, SEMA3F, SEMA4A, SFRP1, SOX17, SHH, FBXL17, SIX3, BMP4, BMP7, BMPR1A, SNAI2, PHACTR4, SMO, SOX5, SOX9, SOX10, SOX11, SRF, STAT3, ZFP36L2, TBXT, TAL1, TBX1, TBX2, TBX5, HNF1B, TCF3, TCF12, TCOF1, TGFB2, TP53, TP73, TWIST1, WNT3, WNT7A, WNT8A, XRCC5, SEMA3B, LIN28A, SETD6, SEMA6D, ELL3, WNT10A, HMGA2, ALX1, LBH, EOMES, FZD1, ITCH, FAM172A, KBTBD8, TEAD2, SEMA7A, RUNX2, RUNX1, CDK13, TP63, CBFB, NRP2, NRP1, ALDH1A2, LDB1, PHOX2B, WNT3A, ACVR1, NOLC1, MYOCD, HAND2, PSMF1, PUM1, SEMA3E, SETD1A, ZEB2, PSMD6 |
| Embryonic diapause score (weight=1) | PDCD4, ACSS1, HEXB, CTSL, RENBP, DPP7, LY6G6E, AUH, HEXA, CCNG2, YPEL2, SPRY1, APOE, ALDH6A1 |
| Embryonic diapause score (weight=-1) | LDHA, PPA1, S100A6, PA2G4, RANBP1, SMS, PHGDH, NAA10, ID3, PLAC8, POLR2I, NUDC, TMEM258, ANAPC15, RAN, ATP5J2, CITED1, MGMT, PRDX2, P4HB, MRPL20, HMGA1, RUVBL2, NDUFC1, MRPL13, HSPE1, MRPS18A, UQCRQ, MRPL18, PSMB3, SEC13, CAPG, GATSL3, TK1, ZCCHC17, PGAM1, RPL41, DAD1, DUSP14, EIF2B2, POLR2L, UCHL1, UQCC2, SLC25A5, PHB, TMEM97, TOMM40L, TIPIN, ARPC1B, PHF5A, SNRPE, SSSCA1, PSMD13, RPS27L, DPH3, RPS19BP1, THOP1, UQCR10, NABP2, CDC34, PELO, CCNE1, FAM118B, SAC3D1, DCTPP1, MDH2, GFER, INO80E, LRRC42, MRPL52, HDLBP, CENPM, YKT6, PSMB6, PMF1, GNG10, PEBP1, CYB5B, EXOSC2, PSMG3, BYSL, ELOF1, ANKRD37, DGCR6, ASB6, CCDC124, RCL1, MRPS11, PSMD14, PRADC1, HILPDA, PSMB4, NDUFS8, MRPL12, TMEM109, UBE2V1, SEPW1, EXOSC4, ATOX1, DRG2, PSMB10, ATP5G1, CETN2, SNRPD2, TMEM39B, TOMM40, PDCD5, EIF3B, CCDC28B, BRMS1 |
| ABC score | ABCA4, ABCB10, ABCD3, ABCC2, ABCC8, ABCG4, ABCC9, ABCD2, ABCB9, ABCC4, ABCD4, ABCA3, ABCC6, ABCC12, ABCC11, ABCC1, ABCA8, ABCA9, ABCA6, ABCA10, ABCA5, ABCC3, ABCA9-AS1, ABCA7, ABCG5, ABCB11, ABCA12, ABCB6, ABCG8, ABCG1, ABCC5, ABCC5-AS1, ABCF3, ABCG2, ABCE1, ABCF1, ABCC10, ABCB4, ABCB1, ABCF2, ABCF2_1, ABCB5, ABCA13, ABCB8, ABCA1, ABCA2, ABCB7, ABCD1 |
| ALDH score | ALDH4A1, ALDH9A1, ALDH18A1, ALDH3B2, ALDH3B1, ALDH1L2, ALDH2, ALDH6A1, ALDH1A2, ALDH1A3, ALDH3A1, ALDH3A2, ALDH16A1, ALDH1L1, ALDH1L1-AS1, ALDH1L1-AS2, ALDH7A1, ALDH8A1, ALDH5A1, ALDH1A1, ALDH1B1 |

**Figure S1**

**
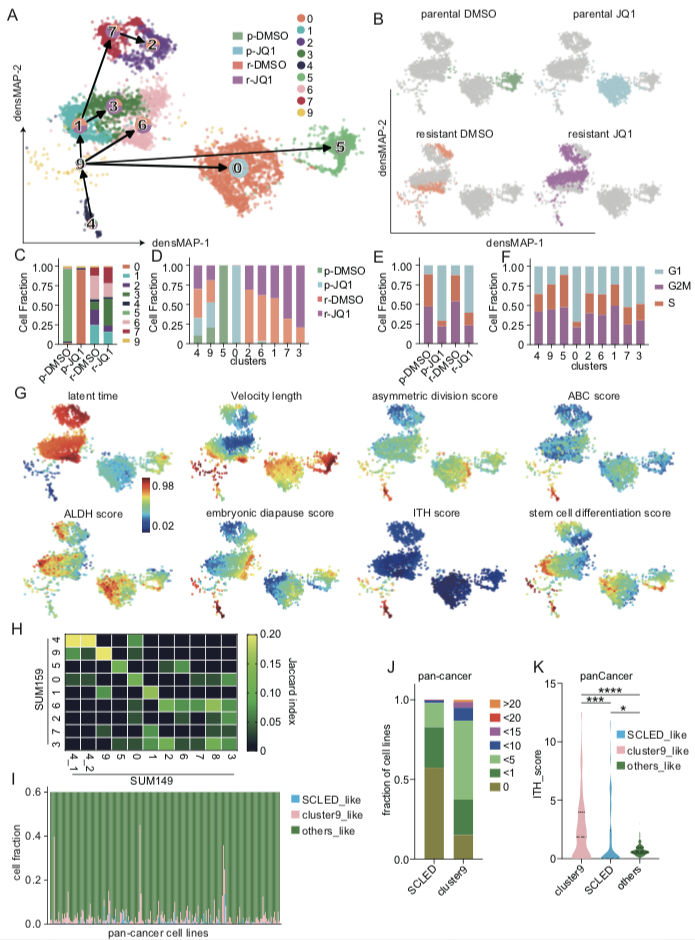
**

**Figure S1, related to Figure 1. SUM159 cells in stem cell-like and embryonic diapause states transit to JQ1 resistant states**

(A) SUM159 were clustered and represented as described in figure 1A. Frequency of cells undergoing different treatments is shown by the pie chart mapped on each cluster. cluster0: n=1207, percentage of mitochondrial reads (pctMT) =7.75. cluster1: n=349, pctMT=9.44. cluster2: n=513, pctMT=6.52. cluster3: n=571, pctMT=8.63. cluster4: n=117, pctMT=42.28. cluster5: n=672, pctMT=6.82, cluster6: n=620, pctMT=11.26. cluster7: n=414, pctMT=6.29. cluster9: n=79, pctMT=14.68.

(B) The origin of each cell from SUM159P and SUM159R and treatment conditions is mapped on the graph in Figure S1A.

(C) Fraction of cells of each cluster in each treatment condition.

(D) Fraction of cells of each treatment condition in each cluster.

(E) Fraction of cells in different cell cycle phases in each treatment condition.

(F) Fraction of cells in different cell cycle phases in each cluster.

(G) Single cells are colored by normalized latent time, velocity length, embryonic diapause score, ABC score, ALDH score, asymmetric division score, ITH score and stem cell differentiation score mapped on to figure 1A. Scale is at the left bottom of the latent time panel.

(H) Heatmap shows Jaccard similarity coefficients between SUM149 and SUM159 clusters using DEGs from each cluster.

(I) Fraction of scPred predicted cluster9-, SCLED-like cells in individual cell lines from GSE157220 pan-cancer dataset.

(J) Fraction of cell lines of pan-cancer cell lines composed by indicated fraction of cluster9-, SCLED-like cells.

(K) Violin plot of ITH-score of cluster9-, SCLED-like and other cells in GSE157220.

**Figure S2**

**
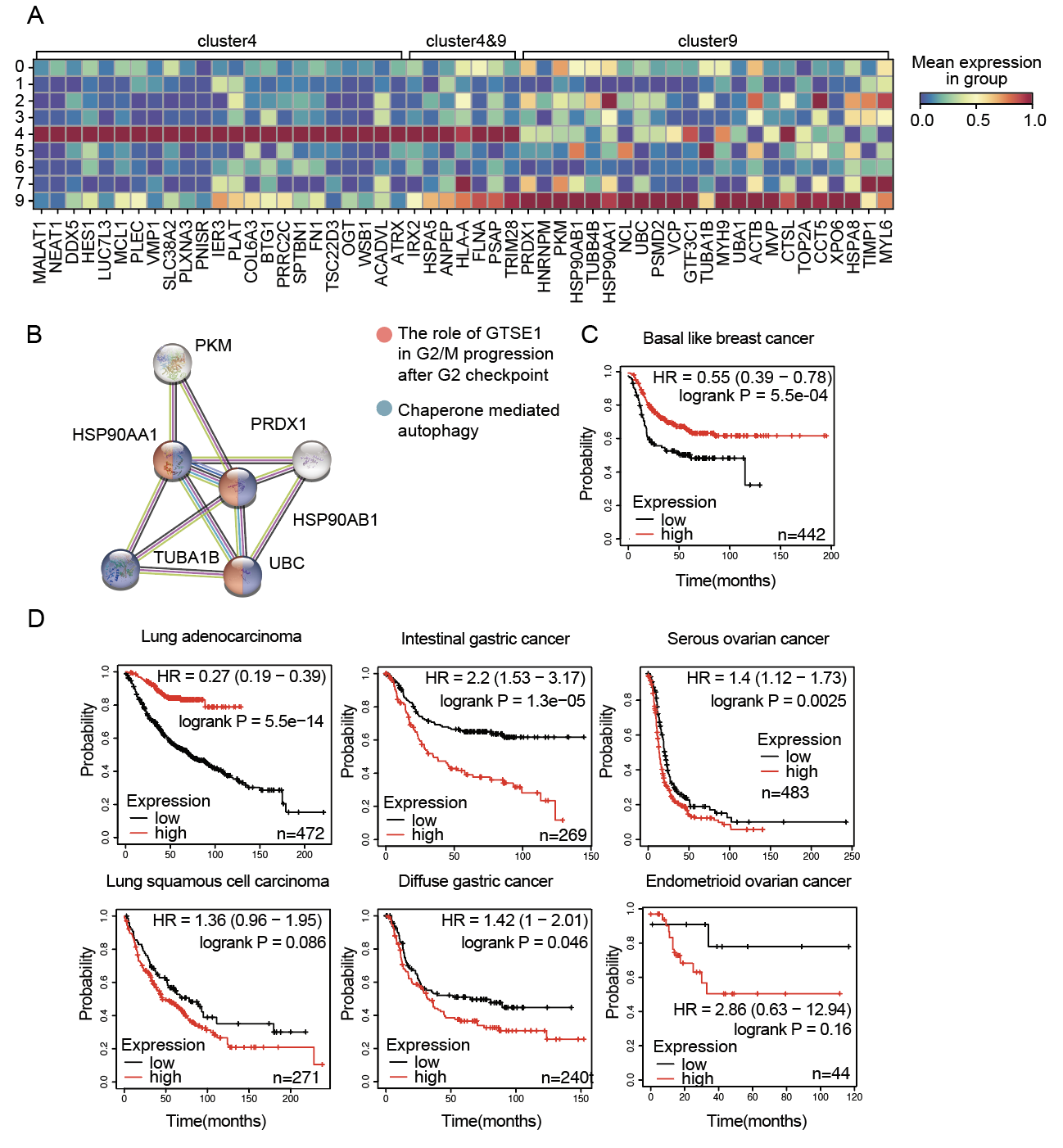
**

**Figure S2, related to Figure 2. Features capturing of embryonic diapause and transitional cell states in SUM159 lines**

(A) heatmap of normalized expression (log_2_(TPM+1)) of SUM159 top 30 differentially expressed genes in indicated clusters.

(B) Protein-protein network of genes overlapping from DEGs of SUM149 cluster 9 and SUM159 cluster 9.

(C) Kaplan-Meier Survival curve shows decreased relapse-free survival in basal-like breast cancer (n=442) patients whose tumors expressed higher levels of embryonic diapause signature. Significance levels were determined by log-rank test.

(D) Kaplan-Meier Survival curve shows controversial prediction of relapse-free survival in patients whose tumors expressed higher levels of embryonic diapause signature in different cancers. Significance levels were determined by log-rank test.

| **Table S2: signature genes of each cluster in SUM149** | | | | | |
| --- | --- | --- | --- | --- | --- |
| cluster0 | cluster4.1 | cluster4.2 | cluster5 | cluster9 | clusterR |
| GAPDH | MALAT1 | MALAT1 | S100A2 | MT2A | RPS6 |
| TMSB10 | NEAT1 | NEAT1 | TMSB10 | KRT81 | MT2A |
| RPS18 | LAMB3 | LAMB3 | AREG | HSP90AA1 | RPS19 |
| RPL12 | HSPA5 | HES1 | RPL37A | S100A6 | RPLP1 |
| S100A2 | LAMA3 | PLEC | YBX1 | ACTG1 | RPL19 |
| NEAT1 | PLEC | VMP1 | RPL35A | GAPDH | TPT1 |
| FTL | HES1 | HSPA5 | RPS29 | PKM | RPS5 |
| TMSB4X | VMP1 | LAMA3 | ATP5ME | LDHB | RPS14 |
| RPL11 | FLNA | F3 | CHCHD2 | ENO1 | RPS4X |
| HMGA1 | HSP90B1 | SAT1 | TMSB4X | TUBB4B | RPS12 |
| S100A10 | KRT19 | ANXA1 | S100A10 | TIMP1 | RPL37 |
| SAT1 | UBC | OGT | RPS21 | PRDX1 | FTL |
| RPS20 | ANXA1 | SLC25A37 | KRT19 | ACTB | RPL32 |
| RPL8 | F3 | FLNA | PTMA | TUBA1B | RPS18 |
| RPL37A | EIF4G1 | EIF4A2 | S100A16 | UBB | RPL36 |
| ACTG1 | PDIA3 | NKTR | RPS7 | HSP90AB1 | RPL29 |
| YBX1 | EIF4A2 | LUC7L3 | RPL38 | TXN | RPS16 |
| ATP5F1E |  | GOLGA8A | ACTB | KRT18 | RPS2 |
| LAMB3 |  | MAT2A | S100A11 | UBC | RPL23 |
| SEC61G |  | ABCA5 | SH3BGRL3 | PSMA7 | RPS27A |
| S100A14 |  | ATRX | H2AFZ | NNMT | RPL34 |
| KRT81 |  | CCNL2 | S100A14 | PSMA4 | RPS15 |
| S100A6 |  | CELF1 | SEC61G | KRT8 | RPLP2 |
| KRT18 |  | PNISR | RPL12 | ANXA2 | RPS15A |
| HES1 |  | FOSB | TXN | PTMA | RPL28 |
| MALAT1 |  | OGA | ATP5F1E | H2AFZ | RPL27 |
| LAMA3 |  | NFKBIZ | GAPDH | LDHA | RPL7A |
| PKM |  |  | CAV1 | TUBB | RPL4 |
| ATP5ME |  |  | HSP90AB1 | NPM1 | RPSA |
| RPL10 |  |  | PSMA7 | HSPA5 | S100A6 |
| RPS8 |  |  | TUBB | AREG | RPS3 |
| ID3 |  |  | HMGB2 | EIF1 | RPL13 |
| S100A16 |  |  | GSTP1 | PGK1 | RPL23A |
| RPS2 |  |  | CAV2 | HSP90B1 | RPS23 |
| PEG10 |  |  | WFDC2 | HSPA8 | RPL31 |
| ANXA2 |  |  | ENO1 | H2AFJ | RPS11 |
| LDHA |  |  | HES4 | ANXA1 | RPL11 |
| ACTB |  |  | ID3 | SEC61G | RPL10 |
| RPL35 |  |  |  | GSTP1 | RPL35 |
| RPL38 |  |  |  | MDH1 | RPL10A |
| RPL35A |  |  |  | PDIA3 | RPL35A |
| HSPA5 |  |  |  | PSMA3 | TIMP1 |
| KRT19 |  |  |  | PCNA | RPL18 |
| SH3BGRL3 |  |  |  | CTSL | RPL14 |
| GSTP1 |  |  |  | CD9 | RPLP0 |
| PRDX1 |  |  |  | HMGB2 | RPL27A |
| TXN |  |  |  | PSMB3 | RPL13A |
| PDIA4 |  |  |  | DNAJA1 | RPS21 |
| RPL10A |  |  |  | S100A11 | RPL37A |
| RPS29 |  |  |  | EPCAM | RPS8 |
| IER3 |  |  |  | CKS2 | UBA52 |
| SQSTM1 |  |  |  | GHITM | RPS7 |
| RPS7 |  |  |  | KPNA2 | RPS24 |
| PLEC |  |  |  | XRCC6 | RPS29 |
| PHLDA2 |  |  |  | S100A10 | RPL5 |
| HSP90B1 |  |  |  | LAPTM4A | RPS20 |
| FLNA |  |  |  |  | EIF1 |
| CYR61 |  |  |  |  | RPL22L1 |
| ID1 |  |  |  |  | ATP5F1E |
| WFDC2 |  |  |  |  | VIM |
| S100A11 |  |  |  |  | LDHB |
| KRT8 |  |  |  |  | RPL8 |
| G6PD |  |  |  |  | MT1X |
| FTH1 |  |  |  |  | MT1E |
| ADIRF |  |  |  |  | TUBA1B |
|  |  |  |  |  | RPL12 |
|  |  |  |  |  | ODC1 |
|  |  |  |  |  | H2AFZ |
|  |  |  |  |  | NNMT |

| **Table S3: signature genes of each cluster in SUM159** | | | | |
| --- | --- | --- | --- | --- |
| cluster0 | cluster4 | cluster5 | clusterR | cluster9 |
| FTL | MALAT1 | TMSB4X | TIMP1 | PKM |
| TPT1 | NEAT1 | RPL29 | RPL13 | PRDX1 |
| GAPDH | IRX2 | RPL19 | MT2A | HSP90AA1 |
| MALAT1 | HES1 | RPS14 | RPS19 | TUBB4B |
| NEAT1 | DDX5 | TUBA1B | RPS5 | HSP90AB1 |
| LGALS1 | HSPA5 | RPL22L1 | RPS3 | IRX2 |
| RPS4X | LUC7L3 | RPL35A | ATP5F1E | NCL |
| S100A6 | VMP1 | BTF3 | RPS6 | FLNA |
| VIM | FLNA | RPL37A | RPS21 | HNRNPM |
| MT2A | HLA-A | RPL23A | RPS16 | PSMD2 |
| TMSB10 | SLC38A2 | RPL32 | RPL12 |  |
| RPL19 | ANPEP | H2AFZ | RPL28 |  |
| SLC25A5 | MCL1 | RPS18 | RPL36 |  |
| RPS12 | PLEC | ACTG1 | RPL37 |  |
| HMOX1 | PSAP | RPSA | PSMA7 |  |
| PKM |  | RPL14 | S100A6 |  |
| PEG10 |  | RPL27 | RPLP2 |  |
| RPL22L1 |  | RPS23 | SH3BGRL3 |  |
| RPS2 |  | CAV1 | GSTP1 |  |
| RPL37A |  | TUBB | RPL18 |  |
| RPS18 |  | RPL11 | RPS7 |  |
| ACTG1 |  | RPS4X | RPL8 |  |
| APOE |  | SLC25A5 | RPLP1 |  |
| RPS20 |  | RPL10 | RPS27A |  |
| RPLP1 |  | RPS12 | RPL7A |  |
| S100A10 |  | RAN | GPX4 |  |
| RPL10 |  | RPL34 | LDHB |  |
| SQSTM1 |  | RPS24 | RPS11 |  |
| TMSB4X |  | HMGB1 | RPS2 |  |
| RPL23A |  | H3F3B | RPL27A |  |
| PRDX1 |  | RPL10A | ANXA2 |  |
| AKR1B1 |  | RPL37 | FTH1 |  |
| RPL23 |  | HSP90AB1 | UBB |  |
| JPT1 |  | RPLP0 | RNASEH2C |  |
| S100A4 |  | RPL38 | S100A16 |  |
| RPL38 |  | PTMA | LDHA |  |
| RPL32 |  | POMP | TPI1 |  |
| RPL14 |  | NCL | TMSB10 |  |
| RPS14 |  | SLC25A6 | RPS15 |  |
| TXNRD1 |  | CYCS | RPL4 |  |
| RPS23 |  | ACTB | ZFAS1 |  |
| MT1X |  | RPL23 | DCUN1D5 |  |
| SLC25A6 |  | RPL31 | CD24 |  |
| ACTB |  | S100A4 |  |  |
| RPL10A |  | GTF3C6 |  |  |
| RHOA |  | NPM1 |  |  |
| ID1 |  | KRT75 |  |  |
| RPS24 |  | YBX1 |  |  |
| POMP |  | TFDP1 |  |  |
| TKT |  | RPS19BP1 |  |  |
| RPL27 |  | SCG5 |  |  |
| H3F3B |  | UBE2S |  |  |
| NPM1 |  | ENO1 |  |  |
| HSP90AA1 |  | SULT1B1 |  |  |
| RPL31 |  |  |  |  |
| TUBB4B |  |  |  |  |
| RPLP0 |  |  |  |  |
| RPL12 |  |  |  |  |
| ID3 |  |  |  |  |
| SPANXB1 |  |  |  |  |
| TGFBI |  |  |  |  |
| RPSA |  |  |  |  |
| ODC1 |  |  |  |  |
| H2AFZ |  |  |  |  |
| TXN |  |  |  |  |
| BTF3 |  |  |  |  |
| LDHA |  |  |  |  |
| ENO1 |  |  |  |  |
| SAA1 |  |  |  |  |
| EIF1 |  |  |  |  |

| **Table S4: results of Cox-regression** | | | | |
| --- | --- | --- | --- | --- |
|  | beta | HR (95% CI for HR) | wald.test | p.value |
| ACTB | 3 | 21 (9.8-43) | 63 | 1.70E-15 |
| ACTG1 | 1.9 | 6.8 (2.6-18) | 15 | 0.00012 |
| ANXA2 | 2.5 | 12 (5.7-27) | 40 | 2.00E-10 |
| CD9 | 3.4 | 31 (15-64) | 82 | 1.20E-19 |
| DNAJA1 | 2.1 | 8 (3.4-19) | 22 | 2.30E-06 |
| ENO1 | 1.5 | 4.7 (1.7-13) | 8.9 | 0.0028 |
| HSP90AA1 | 1.8 | 6 (2.3-16) | 14 | 0.00023 |
| HSP90AB1 | 2.8 | 16 (7.8-33) | 56 | 6.80E-14 |
| KRT8 | 2.7 | 14 (6.3-32) | 41 | 1.60E-10 |
| KRT81 | -14 | 7.5e-07 (0-Inf) | 0 | 0.99 |
| LAPTM4A | 2.5 | 13 (7-23) | 73 | 1.50E-17 |
| LDHA | 1.4 | 4.2 (1.4-13) | 6.3 | 0.012 |
| LDHB | -14 | 7.4e-07 (0-Inf) | 0 | 0.99 |
| MDH1 | 3 | 20 (9.6-43) | 63 | 2.40E-15 |
| NNMT | -14 | 7.5e-07 (0-Inf) | 0 | 0.99 |
| NPM1 | 2.3 | 10 (4.3-24) | 28 | 1.10E-07 |
| PGK1 | 3 | 20 (11-37) | 88 | 6.70E-21 |
| PKM | 2.3 | 10 (4.3-23) | 28 | 1.10E-07 |
| PRDX1 | 2.7 | 15 (7.6-31) | 57 | 3.40E-14 |
| PSMA3 | 2.3 | 10 (4.4-24) | 29 | 7.10E-08 |
| PSMA4 | -0.13 | 0.88 (0.12-6.7) | 0.02 | 0.9 |
| PSMA7 | -14 | 7.4e-07 (0-Inf) | 0 | 0.99 |
| PSMB3 | 1.3 | 3.8 (1.2-11) | 5.5 | 0.019 |
| PTMA | 1.3 | 3.8 (1.2-11) | 5.5 | 0.019 |
| SSBP1 | 1 | 2.9 (0.82-10) | 2.7 | 0.099 |
| TUBA1B | -0.13 | 0.88 (0.12-6.7) | 0.02 | 0.9 |
| TUBB4B | 1.6 | 5.1 (1.8-14) | 9.7 | 0.0019 |
| TXN | 1.5 | 4.4 (1.2-16) | 5.1 | 0.024 |
| UBB | -14 | 7.4e-07 (0-Inf) | 0 | 0.99 |
| UBC | 3.2 | 25 (12-52) | 71 | 3.00E-17 |

**Figure S3**


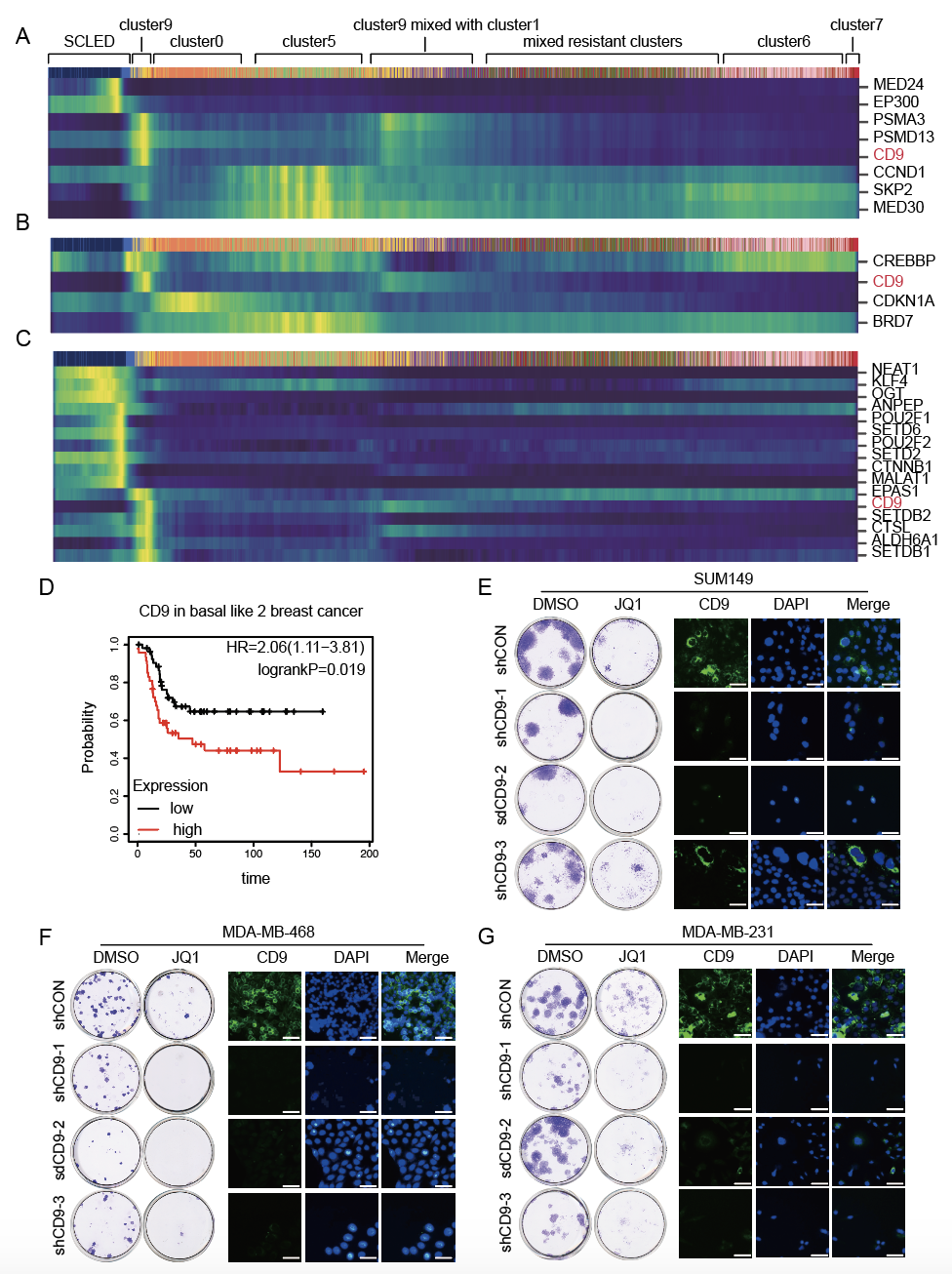


**Figure S3, related to Figure4, shCD9 knockdown mimics increases JQ1 sensitivity in 3 TNBC cell lines**

(A) Heatmap of highly variable genes in our analysis which are synergistic targets from Shu et al study expressed in each individual cell with cells ranked by velocity pseudotime.

(B) Heatmap of highly variable genes in our analysis which are resistant targets from Shu et al study expressed in each individual cell with cells ranked by velocity pseudotime.

(C) Heatmap of highly variable genes in our analysis which are stem cell or embryonic diapause markers from Rehman et al study expressed in each individual cell with cells ranked by velocity pseudotime.

(D) Kaplan-Meier curve of relapse-free survival in patients with basal-like 2 breast cancer (n=101) based on low or high CD9 protein split on median level. Significance is determined by log-rank test.

(E-F) Sensitivity to JQ1 (50nM) for 96h (left) and representative immunofluorescence figures of CD9 surface expression of indicated cell lines. Scale bars: 50 μm.

**Figure S4**


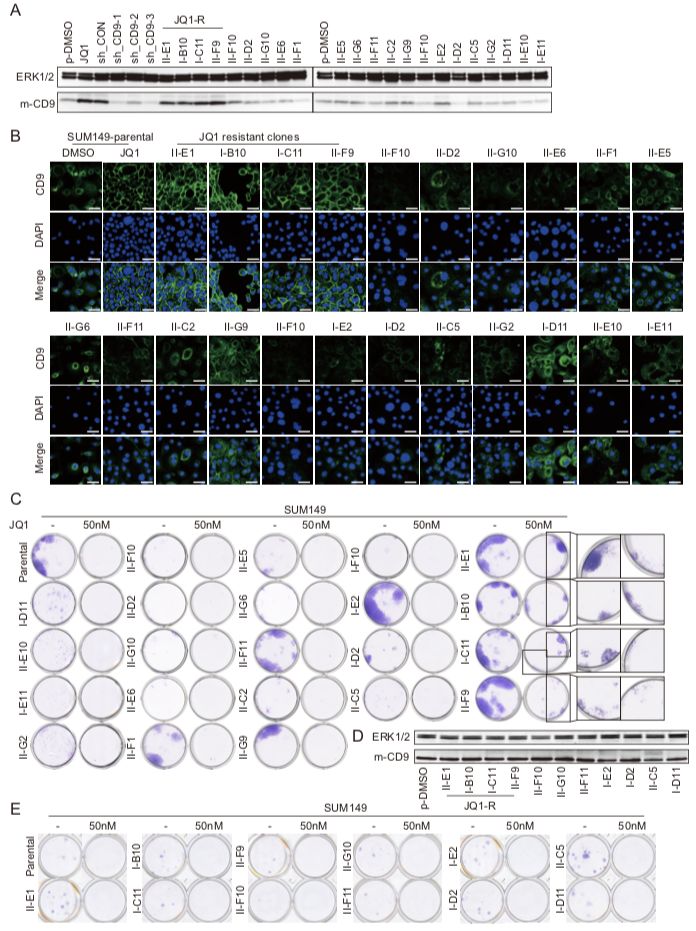


**Figure S4, related to Figure 4. Cell clones selected from SUM149 cell line**

(A) Representative western blot of indicated protein of each clone from SUM149 cell line. Membrane protein was extracted by Subcellular Protein Fractionation Kit for Cultured Cells (#78840, ThermoFisher). Data represents three independent experiments. m-CD9, membrane CD9 expression.

(B) Immunofluorescence images of CD9 expression of each clone from SUM149 cell line. Data is representative of three replicates. Scale bars: 50 μm. CD9 surface expression of SUM149P treated with JQ1, and II-E1, I-B10, I-C11, II-F9 clones was relatively higher than other conditions. II-G9 shows moderate surface CD9 expression. II-D2, II-F1, II-E5, II-G6 and I-D11 show cytoplasm CD9 expression.

(C) Sensitivity of each clone to JQ1 (50nM) for 14 days. Data are representative of one of three replicates.

(D) Representative western blot of CD9 of SUM149 clones after 2-month culture with complete medium. Membrane protein was extracted by Subcellular Protein Fractionation Kit for Cultured Cells (#78840, ThermoFisher). Data are representative of one of three independent experiments. m-CD9, membrane CD9 expression.

(E) Sensitivity of each clone to JQ1 (50nM) for 10 days. Data is representative of one of three replicates.

**Figure S5**


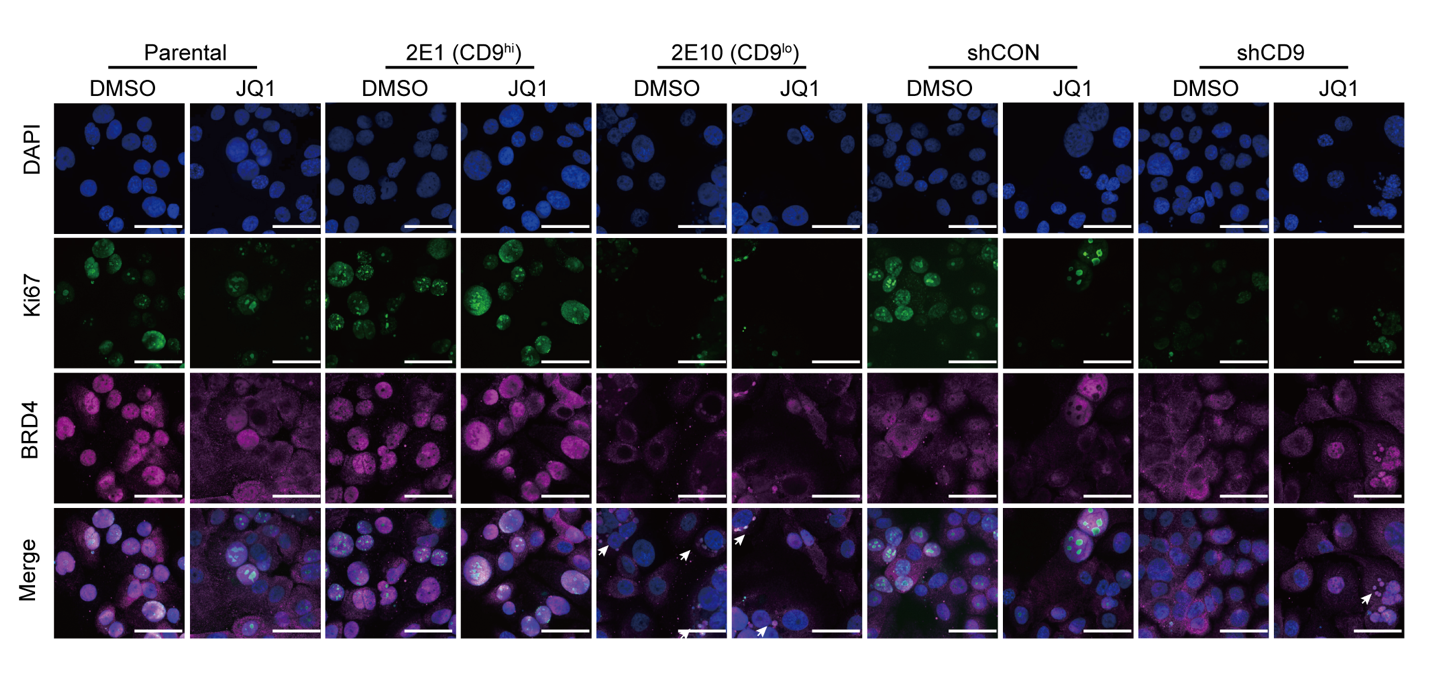


**Figure S5, related to Figure 4. Responses to JQ1 are altered by CD9 expression levels**

Representative immunofluorescence images of response to JQ1 (100nM) for 96 h in the indicated lines. White arrows indicate micronuclei.

**Figure S6**

**
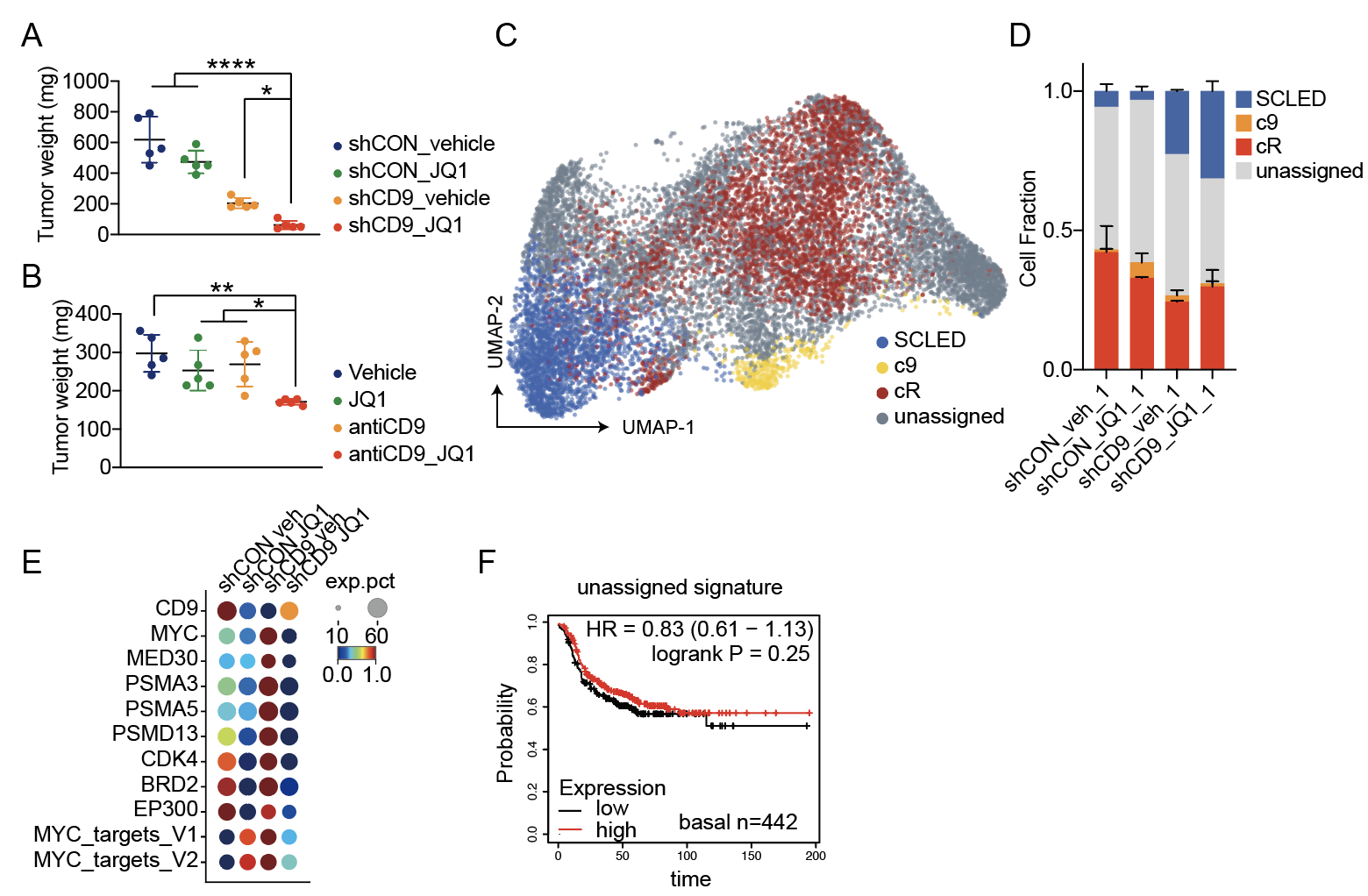
**

**Figure S6, related to Figure 6. Xenograft tumors from Figure 6.**

(A) Tumor weights of indicated groups (n=5) after indicated treatments for 30 days.

(B) Tumor weights of indicated groups (n=5) after indicated treatments for 12 days.

(C) scRNAseq data from tumors at 30 days of (A) was assessed and visualized using UMAP.

(D) Fraction of cells of each assigned cluster in each treatment condition.

(E) Dotplot of highly variable genes which were also reported as synergistic targets in Shu et al’ s study and CD9 and MYC target pathway in indicated treatments.

(F) Kaplan-Meier Survival curve shows no changes of relapse-free survival in basal-like breast cancer (n=442) patients whose tumors expressed higher levels of signature of the assigned cells in xenograft scRNAseq data. Significance levels were determined by log-rank test.

| **Table S5: signature genes of each cluster in xenograft dataset** | | | | |
| --- | --- | --- | --- | --- |
| SCLED | clusterR | cluster9 | unassigned |  |
| SLPI | RPLP1 | S100A9 | TMSB10 |  |
| LCN2 | SPINK6 | S100A8 | S100A2 |  |
| MT-CO2 | MGP | S100A7 | AREG |  |
| MT-ATP6 | KRT17 | PI3 | MT2A |  |
| PI3 | FABP5 | CSTB | ACTG1 |  |
| RARRES1 | RPL10 | SERPINB3 | NEAT1 |  |
| MT-ND4 | TMSB4X | S100A6 | FN1 |  |
| WFDC2 | DSP | LCN2 | VIM |  |
| MT-ND2 | ACTG1 | MT2A | LGALS1 |  |
| LGALS3 | ID3 | CXCL1 | ACTB |  |
| KRT19 | CAST | SERPINB4 | CAV1 |  |
| MMP7 | ID1 | CCL20 | FOS |  |
| MT-ND1 | LMO4 | RARRES1 | SFN |  |
| SAT1 | KRT5 | SAT1 | SH3BGRL3 |  |
| S100A4 | KRT6B | STC1 | PMEPA1 |  |
| CFB |  | SOD2 | TXNDC17 |  |
| KRT81 |  | FTH1 |  |  |
| S100P |  | TNFAIP6 |  |  |
| ZFP36L1 |  | CXCL8 |  |  |
| CTSD |  | MT1X |  |  |
| TNFRSF11B |  | SPINK5 |  |  |
| CSTB |  | SPINK6 |  |  |
| SOSTDC1 |  | TIMP1 |  |  |
| DHCR24 |  | CXCL3 |  |  |
| FGFBP2 |  | TMEM45A |  |  |
| FGFBP1 |  | NDRG1 |  |  |
| MT-ATP8 |  | NAMPT |  |  |
| SLC4A11 |  | CSTA |  |  |
| MT-ND4L |  | SERPINB1 |  |  |
| KRT15 |  | SAA1 |  |  |
| GPRC5A |  | GJB2 |  |  |
| TM4SF1 |  | VEGFA |  |  |
| MIA |  | ERO1A |  |  |
| PSCA |  | PTGS2 |  |  |
| PRKAR2B |  | IGFBP3 |  |  |
| KRT14 |  | STEAP4 |  |  |
| CD24 |  | PDZK1IP1 |  |  |
| CLDN4 |  | SERPINE2 |  |  |
| SPTSSB |  | LDHA |  |  |
| HSPA1A |  | IL1B |  |  |
| SERPINB3 |  | PGK1 |  |  |
| KRT16 |  | S100A7A |  |  |
| FOLR1 |  | CHI3L1 |  |  |
| EHF |  | HBEGF |  |  |
| GSN |  | IFITM3 |  |  |
| ELF3 |  | SCD |  |  |
| PDZK1IP1 |  | BCL2A1 |  |  |
| SERPINB4 |  | S100A12 |  |  |
| FDCSP |  | CSF3 |  |  |
| KRT10 |  | RHCG |  |  |
| PLAAT3 |  | PTPN12 |  |  |
| HLA-A |  | CST6 |  |  |
| IFI27 |  | EFHD2 |  |  |
| ATP1B3 |  | IL36G |  |  |
| FAM3D |  | UPP1 |  |  |
| GABRP |  | CASP14 |  |  |
| MYBPC1 |  | PRSS22 |  |  |
| KRT6B |  | PLA2G4E |  |  |
| NRAD1 |  | SDC4 |  |  |
| CCDC80 |  | AQP3 |  |  |
| SAA1 |  | FGF2 |  |  |
| ALCAM |  | BTG1 |  |  |
| BBOX1 |  | TGM2 |  |  |
| TACSTD2 |  | TMEM158 |  |  |
| AC025580.1 |  | ODAPH |  |  |
| PIGR |  | CDA |  |  |
| AGR2 |  | EREG |  |  |
|  |  | CTSL |  |  |
|  |  | PHLDA2 |  |  |

**Figure S7**

**
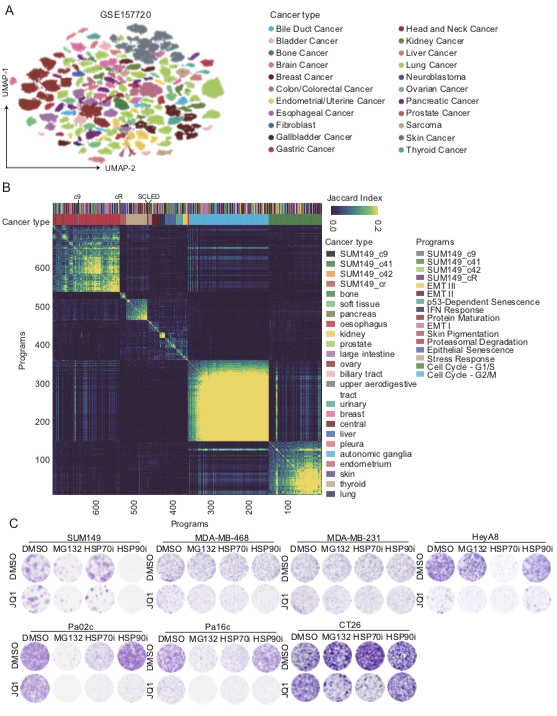
**

**Figure S7, related Figure 3. Synergistic effects of indicated treatments in multiple cancer cell lines in 2D culture.**

(A) Uniform manifold approximation and projection map (UMAP) of 196 cell lines from GSE157220. Each cancer type is indicated by a unique color.

(B)  Heatmap of pairwise similarities between all NMF programs from Kinker et al’s study, ordered by hierarchical clustering.

(C) Sensitivity of each cell line to JQ1 (10nM), MG132 (300nM), HSP70i (5 μM), HSP90i (2.5nM) and the combination of JQ1+MG132, JQ1+HSP70i, JQ1+HSP90i for 14 days. Data are representative of one of three replicates.
